# Implications of genetic variations, differential gene expression, and allele-specific expression on metformin response in drug-naïve type 2 diabetes

**DOI:** 10.1007/s40618-022-01989-y

**Published:** 2022-12-18

**Authors:** M. Vohra, A. R. Sharma, S. Mallya, N. B. Prabhu, P. Jayaram, S. K. Nagri, S. Umakanth, P. S. Rai

**Affiliations:** 1 Department of Biotechnology, Manipal School of Life Sciences, Manipal Academy of Higher Education, Manipal, India; 2 Department of Bioinformatics, Manipal School of Life Sciences, Manipal Academy of Higher Education, Manipal, India; 3 Department of Cell and Molecular Biology, Manipal School of Life Sciences, Manipal Academy of Higher Education, Manipal, India; 4 Department of Medicine, Kasturba Medical College, Manipal Academy of Higher Education, Manipal, India; 5 Department of Medicine, Dr. T.M.A. Pai Hospital, Manipal Academy of Higher Education, Manipal, India

**Keywords:** Allele-specific gene expression, Metformin, RNA-Seq, Targeted exome sequencing, Type 2 diabetes

## Abstract

**Purpose:**

Metformin is widely used to treat type 2 diabetes mellitus (T2DM) individuals. Clinically, inter-individual variability of metformin response is of significant concern and is under interrogation. In this study, a targeted exome and whole transcriptome analysis were performed to identify predictive biomarkers of metformin response in drug-naïve T2DM individuals.

**Methods:**

The study followed a prospective study design. Drug-naïve T2DM individuals (*n* = 192) and controls (*n* = 223) were enrolled. T2DM individuals were administered with metformin monotherapy and defined as responders and non-responders based on their glycated haemoglobin change over three months. 146 T2DM individuals were used for the final analysis and remaining samples were lost during the follow-up. Target exome sequencing and RNA-seq was performed to analyze genetic and transcriptome profile. The selected SNPs were validated by genotyping and allele specific gene expression using the TaqMan assay. The gene prioritization, enrichment analysis, drug-gene interactions, disease-gene association, and correlation analysis were performed using various tools and databases.

**Results:**

rs1050152 and rs272893 in *SLC22A4* were associated with improved response to metformin. The copy number loss was observed in *PPARGC1A* in the non-responders. The expression analysis highlighted potential differentially expressed targets for predicting metformin response (*n* = 35) and T2DM (*n* = 14). The expression of *GDF15, TWISTNB,* and *RPL36A* genes showed a maximum correlation with the change in HbA1c levels. The disease-gene association analysis highlighted *MAGI2* rs113805659 to be linked with T2DM.

**Conclusion:**

The results provide evidence for the genetic variations, perturbed transcriptome, allele-specific gene expression, and pathways associated with metformin drug response in T2DM.

**Supplementary Information:**

The online version contains supplementary material available at 10.1007/s40618-022-01989-y.

## Introduction

Type 2 diabetes mellitus (T2DM) is growing at a distressing rate across the globe, and with-it financial burden on health care is also increasing. T2DM is a complex multifactorial disease with multiple driving factors, including obesity and lifestyle. Lifestyle interventions alone are not always enough for glycaemic control. Metformin is unanimously prescribed by health professionals across the globe as soon as T2DM is diagnosed [1–3]. Metformin is comparatively effective, cheaper, and safe than other anti-diabetic drugs [4, 5]. The initial reports suggest that most individuals tolerate metformin, with ~ 30% showing mild gastrointestinal side effects and ~ 5% of individuals showing severe intolerance [6, 7]. Besides T2DM, metformin is also being used for treatment of polycystic ovarian syndrome (PCOS) [8] and is anticipated to be used in the treatment or prevention of cancer in near future [9].

The proposed mechanism of metformin action includes the mitochondrial complex 1 inhibition, AMPK activation, reduction of cyclic AMP levels, and gut microbe interaction [10–13]. These mechanisms explain the partial benefits of metformin drug and the exact molecular mechanism remains unclear. At present, the guidelines for the treatment of T2DM employ a one-size-fits-all approach without considering inter-individual variation in drug response [14]. Recent literature reports ~ 50% of T2DM fail to reach glycaemic goals after metformin therapy [15]. There are reports of inter-individual variability in metformin response within T2DM that are suggested to be influenced by allelic variants with modest effects [15, 16]. There are several candidate studies evaluating the role of organic cation transporter (OCT) genes and multidrug and toxin extrusion (MATE) genes [17–21]. Multiple variations in *ATM*, *CPA6*, *PRPF31*, and *STAT* genes have also been identified in association with metformin response using genome-wide association studies (GWAS) [22–24]. However, these variations account for a small proportion of variable responses to metformin therapy. Therefore, there is a need to promote the investigations through various omics strategies to develop biomarkers with high therapeutic efficacy [25].

Transcriptome studies have shown that metformin is involved in differential expression of IgA production in the intestinal immune network, cytokine receptor interaction pathways, and genes involved in insulin production and cholesterol homeostasis [26]. The quantitative in vivo evaluation of metformin response revealed both AMPK-dependent and independent networks in the liver [27]. Metabolite analysis has shown significant differences in metabolites such as citric acid, myoinositol, and hippuric acid levels in non-responder and responder groups for metformin drug [28]. A combined metabolome and transcriptome analysis was performed by Udhane et al*.* [29]. They showed that metformin affects glucose, androgen, energy metabolism, immune system, cell cycle, and sex steroid biosynthesis [29]. These evaluations focused on studying the effects of metformin on various genes, pathways, and networks.

We conducted a study on the drug naive T2DM individuals in view of highlighting the genetic and transcriptomic features that may influence the metformin treatment. In this study, next-generation sequencing (NGS) [30] was utilized to analyze the genetic variations and transcriptomic alterations, to elucidate the allele-specific expression and its implications on clinical response to metformin in drug-naïve T2DM individuals.

## Methods

### Ethics statement

**Fig. 1:**
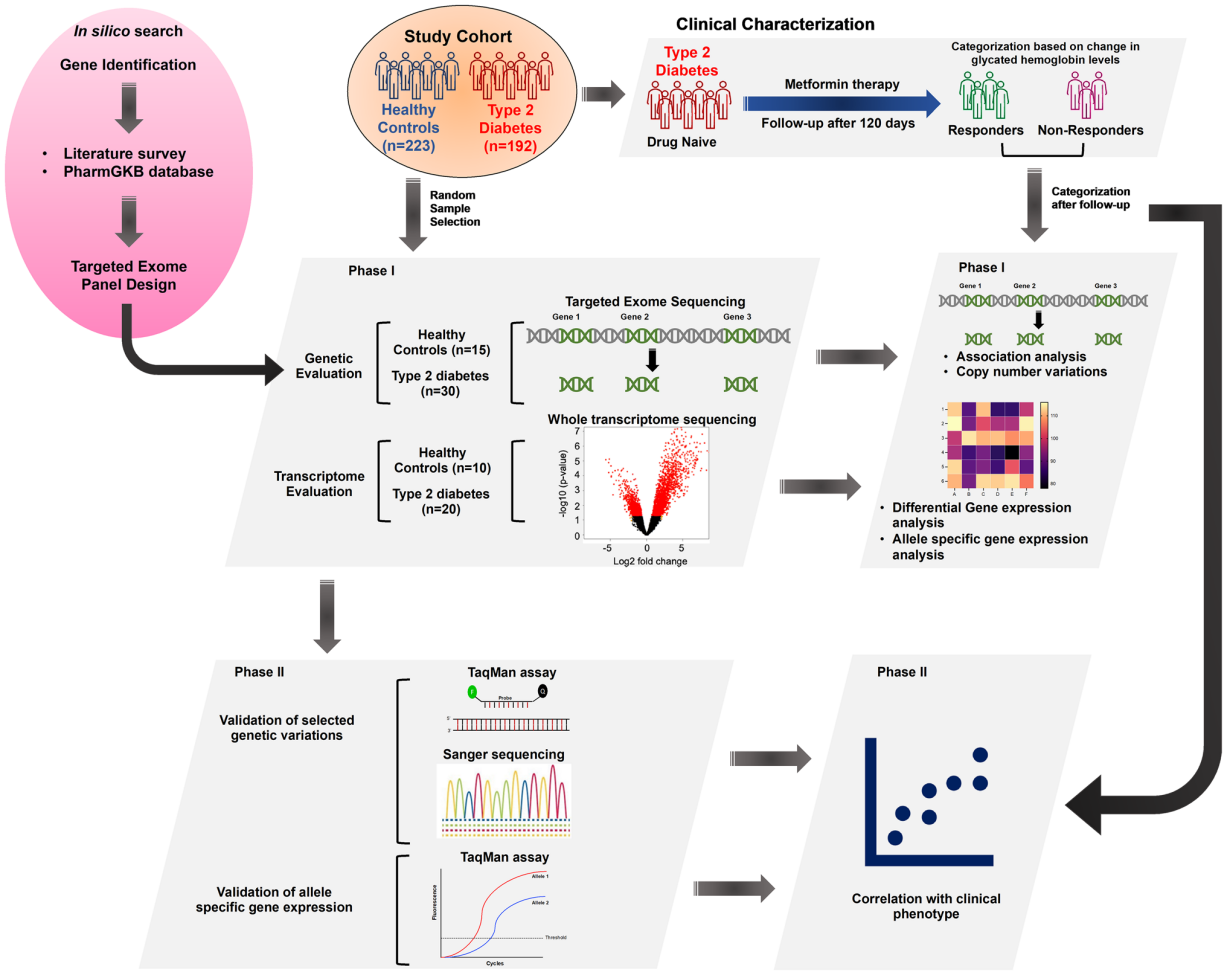
A detailed flow chart depicting the workflow of the study

The protocols and work conducted in this study were in accordance with the declaration of Helsinki. The ethical approval for conducting the study was obtained from Institutional Ethics Committee, Kasturba Hospital, Manipal, India file number: IEC 501/2016. The study was also registered under the Clinical Trials Registry-India (CTRI) (registration number CTRI/2018/01/011508). Written informed consent was obtained from all participants after explaining the study procedure. Further, the work plan of the current study is portrayed in Fig. 1.

### Study subjects

The participants were enrolled from the outpatient department of Dr. TMA Pai Hospital, Udupi, and Kasturba Hospital, Manipal, India. T2DM was defined based on WHO criteria. The details are presented in the supplementary information. A total of 192 T2DM and 223 healthy participants were enrolled. The glycaemic index of the T2DM participants was recorded at the time of enrolment and post metformin monotherapy of 3 months. The response to metformin was defined as the decrease in glycated haemoglobin (HbA1c) level > 1% or > 20 mg/dl decrease in fasting blood glucose level from baseline after three months of therapy. The blood samples (5 ml) were used for isolation of DNA and RNA based on the standard protocols [31].

### Targeted exome sequencing

The targeted exome sequencing was performed on Ion torrent PGM platform (ThermoFisher Scientific, US) for the genes selected from the pharmacokinetic and pharmacodynamic pathways of metformin in PharmGKB database (Supplementary Table S1). The variants identified from each sample were further grouped based on responder, non-responder and healthy control status, and compared using Maftools with default settings [32]. The copy number analysis was performed using CNVKit (v0.9.2) with default settings [33]. The detailed method for targeted exome sequencing and analysis is presented in supplementary information. We hypothesized that changes in genetic profile may predict the metformin drug response status of a T2DM individual.

### Whole transcriptome sequencing

Whole transcriptome sequencing was performed on 30 RNA samples using poly(A) enrichment of the mRNA using dynabeads mRNA direct micro kit (Invitrogen, US; Thermo Fisher Scientific, Inc., US). The detailed method for whole transcriptome sequencing and analysis is presented in supplementary information. We hypothesized that variations in the gene expression may predict the metformin drug response status of a T2DM individual.

### Functional analysis and clinical correlation

The gene ontology analysis was performed on differentially expressed genes (DGE) using Enrichr (http://amp.pharm.mssm.edu/Enrichr/) [34]. The enrichment of bio-pathways through KEGG pathways was confirmed with Cytoscape plugin ClueGO + Cluepedia app [35, 36]. The DGEs were evaluated using DGIdb database (source: http://dgidb.org/, assessed on 01 May 2020).

To identify the relationship between the gene expression levels and metformin response, we performed a Pearson’s correlation analysis between the change in HbA1c levels after 3 months of therapy and the gene expression profiles in responders and non-responders.

### Identification of SNPs in the DGE

The list of DGEs after enrichment analysis was subjected to analysis using DisGeNET database [37]. The genes were investigated for associations with diabetes or other diseases. The gene names were used as identifiers and the variations listed in the Evidences for Variant-Disease Associations column were extracted. The R package i.e., networkD3 was used for the visualization of associations of the variants in the genes associated with different diseases.

### SNP genotyping and Sanger sequencing

The high frequency SNPs selected from ASE analysis along with two coding SNPs namely rs1050152 and rs272893 in *SLC22A4* gene were subjected to genotyping using TaqMan Assay. The DNA samples were randomly validated using Sanger sequencing. The rs1050152 and rs272893 in *SLC22A4* gene were selected based on the association analysis.

### Allele specific expression (ASE) analysis

The ASE analysis was performed on the RNA samples identified as heterozygous for selected SNPs. The RNA samples were converted into cDNA using High-Capacity cDNA Reverse Transcription Kit (Applied Biosystems, US). The cDNAs were used to conduct TaqMan assay using custom designed probes from Custom TaqMan Assay Design Tool (Thermo Fisher Scientific, US) for cDNA samples. The ratio of VIC (mutant allele) and FAM (wild allele) was log transformed and evaluated for differences between responders and non-responders.

### Statistical analysis

The difference between the quantitative variables of the study groups was assessed using student’s *t*-test and ANOVA. The association analysis of SNPs with metformin drug response was evaluated using SNPstats at *p*-value threshold of 0.05. The difference in allele specific expression between responders and non-responders was evaluated using student’s *t*-test.

## Results

### Study participants

We enrolled a total of 192 T2DM and 223 healthy participants (Table 1). During the study course, 19 participants in the T2DM group discontinued the follow-up, 15 participants discontinued the drug, and 12 participants switched to alternative medicine or therapies such as ayurvedic medicine, homeopathy, etc. The final analysis was performed on 146 T2DM individuals who completed three months of follow-up. Among the 146 T2DM participants, 82 were considered responders, and 64 were considered non-responders based on our criteria.

**Table 1:** Clinical characteristics of the study participants enrolled in the study

Clinical characteristics	Type 2 diabetes (*n* = 192) †		Controls (*n* = 223)	*p*-value
	Responders (*n* = 82) ‡	Non-responders (*n* = 64) ‡		
Demographs				
Age (years)	51.9 ± 8.6		50.63 ± 9.1	0.14
	46.97 ± 17.17	49.11 ± 14.71		0.4
Gender (male/female)	139/53		157/66	**–**
	51/31	45/19		**–**
Anthropometric measurements				
BMI (kg/m^2^)	26.09 ± 5.5		22.04 ± 1.4	< 0.0001*
	24.28 ± 6.83	26.93 ± 4.57		0.01
Weight (kgs)	69.79 ± 13.57		55.49 ± 19.23	< 0.0001*
	69.03 ± 10.57	70.64 ± 12.54		0.41
Waist circumference (cms)	99.69 ± 9.89		82.44 ± 8.94	< 0.0001*
	89.57 ± 32.15	95.73 ± 25.23		0.22
Glycaemic Index				
HbA1c (%)	8.26 ± 1.68		5.4 ± 0.38	< 0.0001*
	7.44 ± 1.67	7.59 ± 2.81		0.69
Fasting glucose (mg/dl)	171.21 ± 56.57		93.4 ± 6.88	< 0.0001*
	127.9 ± 63.96	134 ± 90.82		0.64
Post prandial glucose (mg/dl)	226.89 ± 86.73		108.6 ± 12.46	< 0.0001*
	179.61 ± 121.6	199.33 ± 111.73		0.33
Lipid profile				
Total cholesterol (mg/dl)	198.21 ± 58.83		149.32 ± 36.2	< 0.0001*
	144.54 ± 84.51	161.47 ± 91.66		0.26
Triglycerides (mg/dl)	180.91 ± 95.55		120.41 ± 25.4	< 0.0001*
	149.09 ± 124.4	139.4 ± 113.01		0.26
HDL cholesterol (mg/dl)	39.92 ± 14.11		46.44 ± 19.2	< 0.0001*
	30.61 ± 17.81	33.3 ± 12.88		0.32
LDL cholesterol (mg/dl)	126.24 ± 51.02		86.4 ± 10.03	< 0.0001*
	70.99 ± 47.55	140.57 ± 50.76		< 0.0001*

All continuous variables are expressed as mean ± SD

*BMI* body mass index, *HbA1c* glycated hemoglobin, *HDL* high density lipoprotein, *LDL* low density lipoprotein

*p-value< 0.05 was considered statistically significant

**†**Total number of enrolled study participants

**‡**Participants who followed 3 months of follow-up

### Polymorphism spectrum in study subjects

**Fig. 2:**
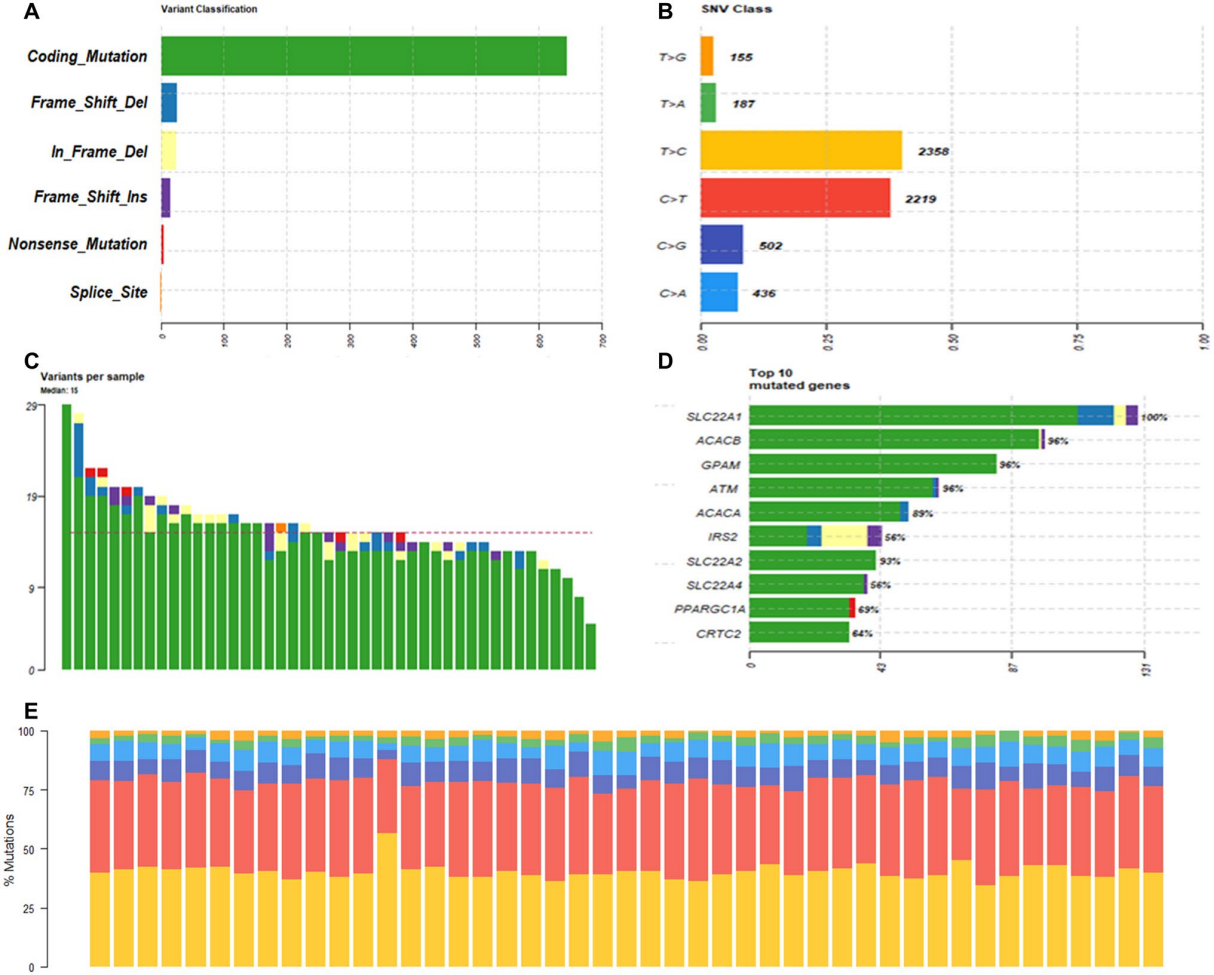
Summary statistics of the targeted exome sequencing **a** variant classification **b** SNV class **c** variants per sample **d** top 10 mutated genes **e** percentage of mutations in the samples

The targeted sequencing of 22 genes was performed in drug naïve T2DM individuals (*n* = 30) and controls (15). On average, 192,652 reads were generated from each T2DM sample, and 206,153 reads were generated from each control sample (Supplementary Table S2). The average percentage quality score of Q20 was observed for an average of 88.8% ± 1.35 and 94.48% ± 0.22 for T2DM and control samples, respectively. The total variants identified in 45 samples from our bioinformatics analysis were 715 in 22 genes (Supplementary Table S3). The maximum number of variations were identified in the* SLC22A1* gene, and the least number of variations were identified in the *ABCC8* gene (Supplementary Table S3). The summary of variations identified from our analysis is shown in Fig. 2. The transitions were higher than transversions observed in our data in all subjects.

### Association analysis for drug response

**Fig. 3:**
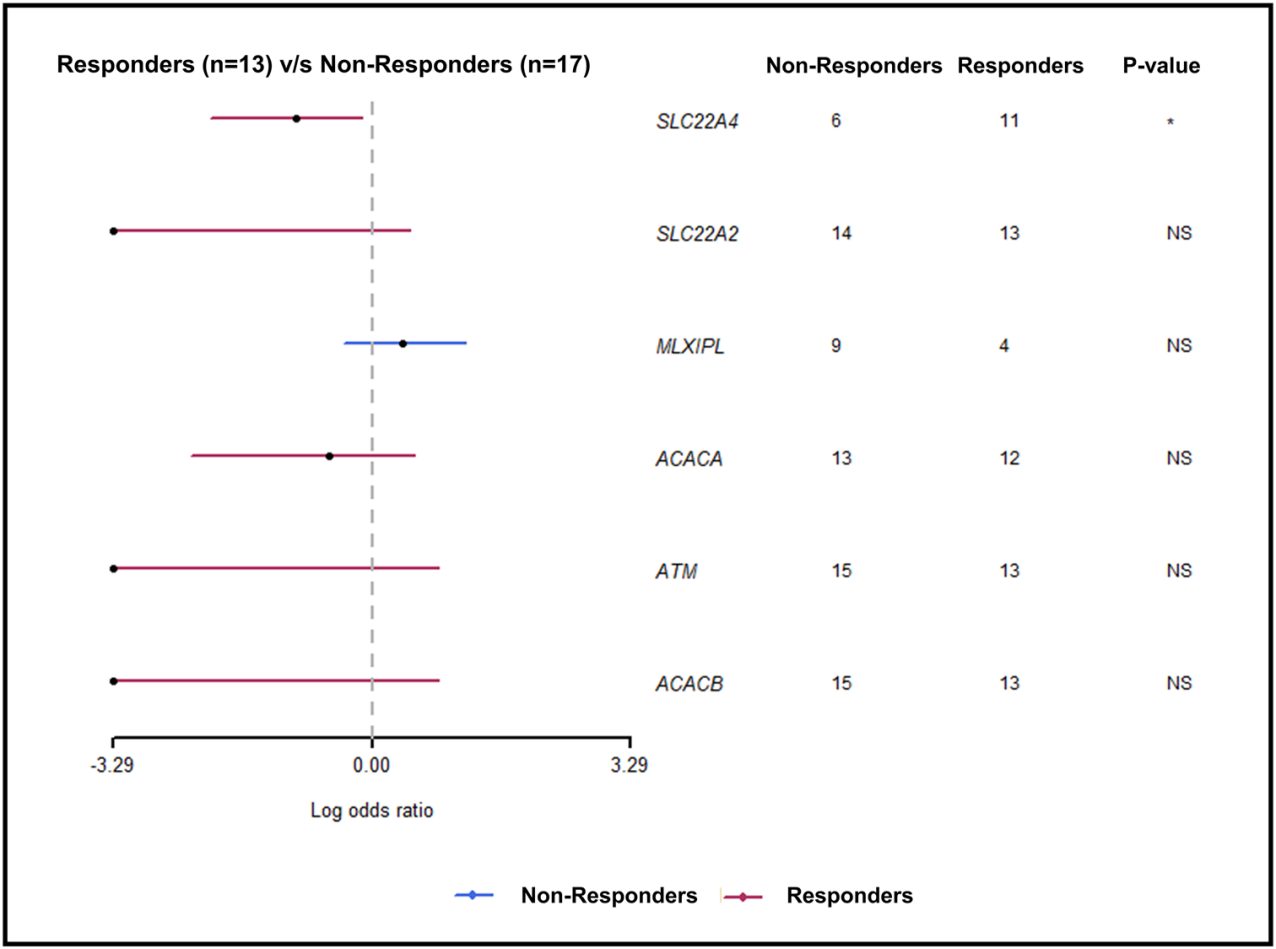
Association analysis between responders and non-responders using maftools

To identify the genetic variations associated with drug response, we compared non-responders (*n* = 17) vs responders (*n* = 13) using MAFtools pipeline at default settings (Fig. 3). The analysis highlighted top mutated genes between responders and non-responders. A significant association of metformin drug response was observed for variations in the *SLC22A4* gene with an odds ratio of 0.1 (0.008–0.73) with a *p*-value of 0.01 (Supplementary Table S4). From the association analysis conducted using MAFtools, we identified top mutated genes in responders and non-responders. Further using manual search, we identified the presence of rs272893 variant in 11 responder samples and 5 non-responder samples and rs1050152 variant in 8 responder samples and 1 non-responder sample. These two variants, rs272893 and rs1050152, were selected for replication in the study cohort.

### Copy number variants in drug response genes

**Fig. 4:**
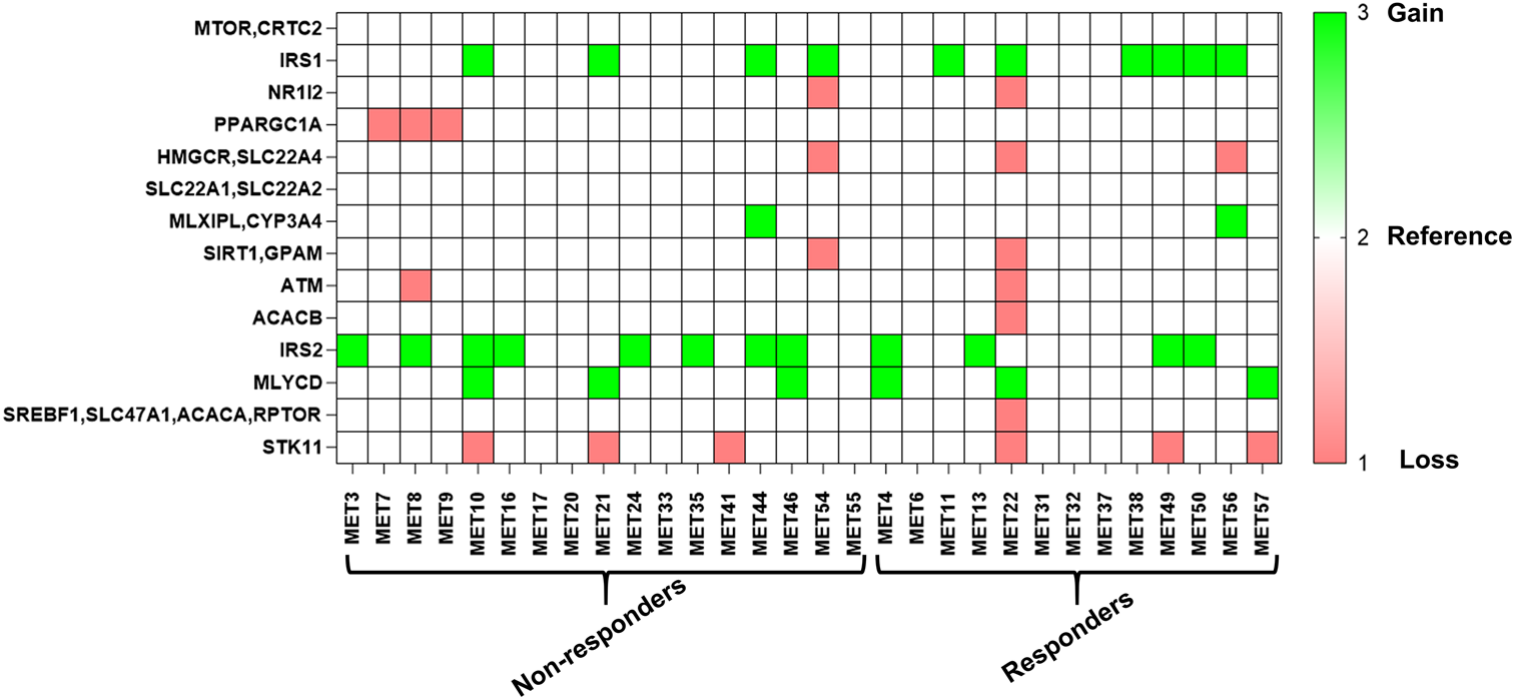
Copy number variations observed in responders and non-responders using CNVKit

The comparison with the baseline for the copy number variations in responders and non-responders samples revealed a unique pattern of copy number gains and losses (Fig. 4) (Supplementary Table S5). *IRS1* was found to have copy number gain in 23.5% of non-responders and 46.15% of responders. *IRS2* was found to have copy number gain in 47.05% of non-responders and 30.76% of responders. *PPARGC1A* was found to have a loss of copy number in 17.6% of non-responders, whereas no copy number loss was observed in the responder samples. *STK11* was found to have copy number loss in 17.6% of the non-responders and 23.07% of responders.

### Characterization of gene expression in metformin responders, non-responders, and healthy controls

The RNA-Seq data generated from metformin responders (*n* = 10), non-responders (*n* = 10) and healthy controls (*n* = 10) showed 55,765 raw features across 30 samples (Supplementary Fig. 1). After initial filtering and data normalization, we obtained 15,434 features across 30 samples (Supplementary Fig. 1). The comparison between three groups was made in combinations such as non-responders vs. responders, responders vs. healthy controls, and non-responders vs. healthy controls. The results are presented in the form of Volcano plots (Supplementary Fig. 2). Using the criteria i.e., FC > 2 (upregulated) or < -2 (downregulated), FDR < 5% and *p*-value < 0.05, we found 165 protein-coding genes in non-responders vs responders, 435 protein-coding genes in responders vs healthy controls, and 257 protein-coding genes in non-responders vs healthy controls. Out of these genes, 38 protein-coding genes in non-responders vs. responders, 405 protein-coding genes in responders vs. healthy controls and 237 protein-coding genes in non-responders vs. healthy controls were upregulated, and 127 protein-coding genes in non-responders vs. responders, 30 protein-coding genes in responders vs. healthy controls and 20 protein-coding genes in non-responders vs. healthy controls were downregulated (Supplementary Fig. 3).

### Gene prioritization and enrichment analysis

For gene prioritization, we evaluated the overlapping upregulated and downregulated protein-coding genes in non-responders vs. responders, responders vs. healthy controls, and non-responders vs. healthy controls (Supplementary Table S6). We observed 29 protein-coding genes upregulated in non-responders when compared with responders or healthy controls (DR_Set_1), 59 protein-coding genes upregulated in both non-responders and responders when compared to healthy controls (T2DM_Set_1), 11 protein-coding genes downregulated in both non-responders and responders when compared healthy controls (T2DM_Set_2) and 105 protein-coding genes upregulated in responders when compared with healthy controls but downregulated in non-responders when compared with responders (DR_Set_2) (Supplementary Table S6).

The genes in the above-mentioned four groups/lists were considered for enrichment analysis and the top 30 biological processes, molecular function, and cellular components, along with their corresponding genes are presented in Supplementary Figs. 4, 5, 6, 7. The network analysis of the selected enriched genes from DR_Set_1 showed pathways such as Ras signalling, tryptophan metabolism, neuroactive ligand-receptor interaction, Hippo signalling, nicotinate, and nicotinamide metabolism, glycine, serine, and threonine metabolism (Supplementary Fig. 4). The network analysis of the selected enriched genes from T2DM_Set_1 showed pathways such as oxidative phosphorylation, Wnt signalling, MAPK signalling, miRNAs in cancer, PI3K-Akt signalling, TGF-beta signalling, cytokine–cytokine receptor interaction, viral myocarditis, ubiquitin-mediated proteolysis (Supplementary Fig. 5). The network analysis of the selected enriched genes from T2DM_Set_2 showed pathways such as NOD-like receptor signalling, lysosome receptor protein, cytokine–cytokine receptor interaction, neuroactive ligand-receptor interaction (Supplementary Fig. 6). The network analysis of the selected enriched genes from DR_Set_2 showed pathways such as Wnt signalling, hippo signalling, calcium signalling, p53 signalling, thyroid hormone signalling, MAPK signalling, RNA polymerase, RNA transport, protein export, spliceosome, ribosome, purine metabolism, retinol metabolism, lipoic acid metabolism, arginine and proline metabolism, amino sugar and nucleotide sugar metabolism, steroid hormone biosynthesis, cytokine–cytokine receptor interaction, bacterial invasion of epithelial cells, vasopressin- regulated water reabsorption, African trypanosomiasis.

### DEGs in metformin drug response

The prediction of metformin drug response from the patient’s gene expression profile required comparing responders, non-responders, and healthy controls. From the comparisons, we observed that 29 genes were significantly upregulated in non-responders compared with responders and healthy controls (DR_Set_1). We also observed 105 genes to be upregulated in responders compared to healthy controls and downregulated in responders when compared with non-responders (DR_Set_2). Thus, genes from gene set 1 were considered for predicting non-responder’s status and gene set 4 for prediction of responder’s status.

**Fig. 5:**
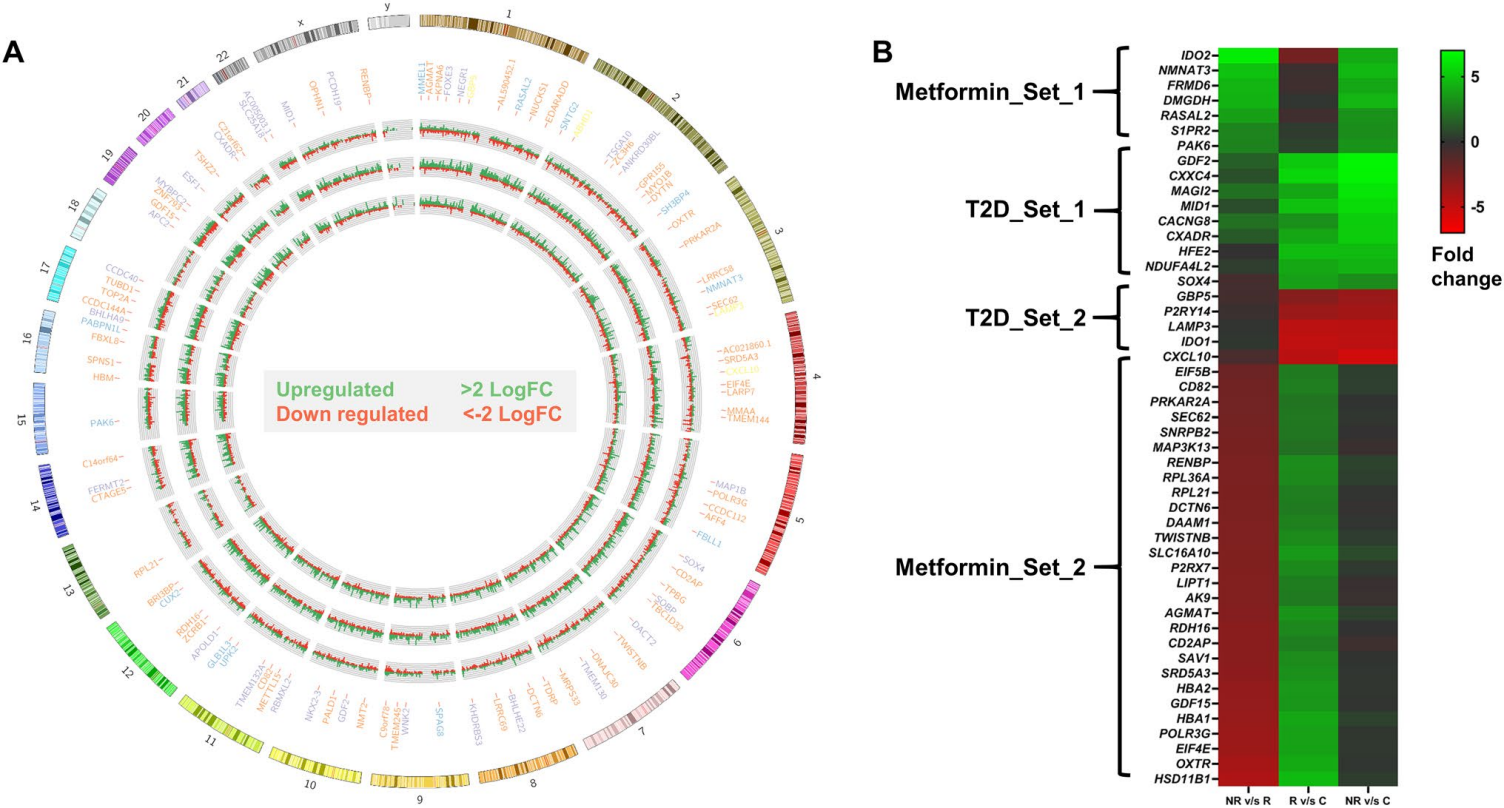
Differentially expressed genes in the study population. **a** Circos plot showing the differential gene expression across the human genome. Tracks from outer to inner represent: Gene names with FC > 2 (upregulated) or < − 2 (downregulated), FDR < 5% and *p*-value < 0.05 (Light Blue, Metformin_Set_1; Purple, T2D_Set_1; Yellow, T2D_Set_2; Orange, Metformin_Set_2); log2FC between Responders vs Control; Non-responders vs Control and Non-responders vs Responders. **b** Heatmap showing the log2FC of enriched genes in Non-responders (NR) vs Responders (R), Responders (R) vs Control (C) and Non-responders (NR) vs Control (C)

Out of 29 genes from DR_Set_1, seven genes were enriched from the gene ontology and KEGG pathway analysis (Supplementary Table S7) (Fig. 5). *IDO2*, *NMNAT3*, *FRMD6*, and *DMGDH* genes from gene set 1 showed maximum log fold change > 4. These genes were selected after enrichment and were shown to be involved in tryptophan metabolism, nicotinate and nicotinamide metabolism, Hippo signalling, and glycine, serine, and threonine metabolism, respectively.

Out of 105 genes from DR_Set_2, 28 genes were enriched from the gene ontology and KEGG pathway analysis (Supplementary Table S7) (Fig. 5). Among these genes, only the *HSD11B1* gene showed the maximum log fold change i.e., > 4. *HSD11B1* gene played a role in steroid hormone biosynthesis, chemical carcinogenesis and metabolism of xenobiotics by cytochrome P450.

The genes enriched from DR_Set_1 (*n* = 7) and DR_Set_2 (*n* = 28) were evaluated for drug-gene interactions. A total of 8 genes were found to interact with 25 different drugs (Supplementary Table S8). Among these eight genes, *the NMNAT3* gene belonged to DR_Set_1, whereas *RENBP*, *LIPT1*, *GDF15*, *HBA1*, *EIF4E*, *OXTR*, and *HSD11B1* belonged to DR_Set_2. None of these genes showed interactions with diabetes drugs.

The correlation analysis between metformin response and gene expression profiles of responders and non-responders highlight the direct correlation of *CD82* (*r* = 0.4, *p*-value = 0.04), *DCTN6* (*r* = 0.63, *p*-value = 0.002), *EIF4E* (*r* = 0.68, *p*-value = 0.0008), *GDF15* (*r* = 0.79, *p*-value < 0.0001), *HBA2* (*r* = 0.45, *p*-value = 0.04), *LIPT1* (*r* = 0.64, *p*-value = 0.002), *MAP3K13* (*r* = 0.49, *p*-value = 0.02), *PRKAR2A* (*r* = 0.58, *p*-value = 0.007), *RENBP* (*r* = 0.61, *p*-value = 0.004), *RPL36A* (*r* = 0.71, *p*-value = 0.0004) and *TWISTNB* (*r* = 0.73, *p*-value = 0.0002) with change in HbA1c levels after 3 months of metformin therapy (Supplementary Fig. 8).

### DGEs in T2DM

From the comparisons made between responders, non-responders, and healthy controls, we observed two gene sets, i.e., T2DM_Set_1 and T2DM_Set_2, with DGEs when responders or non-responders were compared with healthy controls (Fig. 5B). The genes from T2DM_Set_1, namely, *CXXC4, GDF2, MID1,* and *NDUFA4L2*, were found to be upregulated with a log fold change > 4 in both responders and non-responders when compared with controls. These were involved in Wnt signalling, cytokine–cytokine receptor interaction, ubiquitin-mediated proteolysis, and oxidative phosphorylation pathways as predicted by Kegg pathways. The genes from T2DM_Set_2, namely, *IDO1, LAMP3*, and *CXCL10*, were downregulated with a log fold change value < − 4 in both responders and non-responders when compared with controls and were involved in tryptophan metabolism, lysosome, and cytokine–cytokine receptor interaction pathways.

### SNPs in the DGEs associated with diabetes

40 SNPs were identified in 6 out of 14 DGEs T2DM_Set_1 and T2DM_Set_2 with validated associations with 20 different disease conditions or traits (Supplementary Fig. 9). Among these associations, rs113805659 in the *MAGI2* gene was associated with non-insulin-dependent diabetes mellitus in African Americans [38].

### Allele specific expression analysis (ASE)

**Fig. 6:**
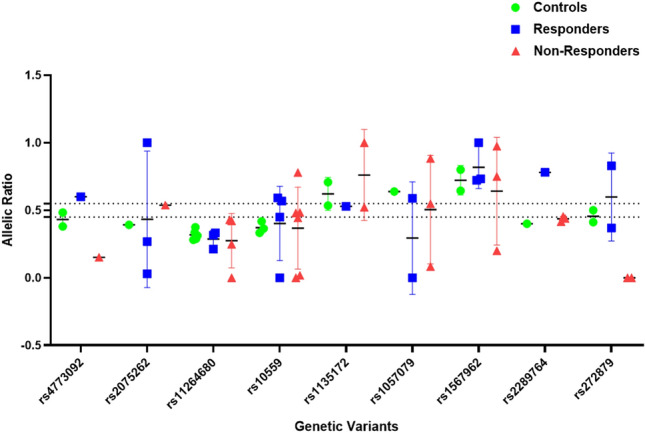
SNPs showing allele specific gene expression from the NGS data

The ASE was observed for 9 variants with high frequency namely, *IRS2* rs4773092, *ACACB* rs2075262, *CRTC2* rs11264680, *CRTC2* rs10559, *MTOR* rs1135172, *MTOR* rs1057079, *RPTOR* rs1567962, *RPTOR* rs2289764, and *SLC22A4* rs272879 (Fig. 6) (Supplementary Table S9). Interestingly, in non-responder samples heterozygous for *SLC22A4* rs272879, the wild allele was only was expressed (the allelic ratio was 0) compared to responder and control samples where both wild and mutant alleles were expressed (the allelic ratio was 0.6 and 0.46, respectively). These 9 variations were selected for primer design using TaqMan assay's custom probes. Out of 9 variations, the custom probes for 5 variants namely *ACACB* rs2075262, *MTOR* rs1135172, *MTOR* rs1057079, *RPTOR* rs2289764, and *SLC22A4* rs272879.

### Association analysis

The association analysis for metformin drug response was performed between responders (*n* = 82) and non-responders (*n* = 64). The association analysis was performed for five variants selected from ASE analysis and two variants selected from targeted exome data (Table 2). Out of the seven variants, the association of *SLC22A4* rs1050152 and *SLC22A4* rs272893 was significant for metformin drug response (Table 2). The *SLC22A4* rs1050152 showed an odds ratio of 0.6 (0.3–1.002), with *p*-value of 0.04 whereas *SLC22A4* rs272893 showed an odds ratio of 0.47 (0.2–0.8), with *p*-value of 0.005. The Sanger sequencing performed on the randomly selected samples showed 100% concordance with the NGS and genotyping data.

**Table 2:** The association analysis of selected SNPs with metformin response

Gene SNPID	Genotype allele	Responders (*N* = 82)	Non-responders (*N* = 64)	OR (95% CI) *p*-value	HWE
*ACACB* rs2075262	G/G	49	38	0.9 (0.5–1.5) 0.75	0.001*
	G/A	21	19		
	A/A	12	7		
	G-allele	119	95		
	A-allele	45	33		
*MTOR* rs1135172	A/A	46	30	1.2 (0.7–2.07) 0.4	0.9
	G/A	31	28		
	G/G	5	4		
	A-allele	123	88		
	G-allele	41	36		
*MTOR* rs1057079	C/C	41	33	1.1 (0.6–1.8) 0.5	0.7
	C/T	33	20		
	T/T	8	11		
	C-allele	115	86		
	T-allele	49	42		
*PIK3CD* rs11121484	C/C	39	26	1.2 (0.75–1.9) 0.41	0.009*
	C/T	27	24		
	T/T	16	14		
	C-allele	105	76		
	T-allele	59	52		
*PIK3R1* rs706713	C/C	40	25	1.4 (0.8–2.2) 0.15	0.02*
	C/T	28	24		
	T/T	14	15		
	C-allele	108	74		
	T-allele	56	54		
*PIK3R5* rs394811	G/G	37	29	0.96 (0.5–1.5) 0.88	0.17
	G/A	40	32		
	A/A	5	3		
	G-allele	114	90		
	A-allele	50	38		
*RPTOR* rs2289764	T/T	47	31	1.3 (0.8–2.2) 0.23	0.6
	T/C	29	26		
	C/C	6	7		
	T-allele	123	88		
	C-allele	41	40		
*SLC22A4* rs1050152	C/C	33	36	**0.6 (0.3–1.002)** 0.04*	0.3
	C/T	41	28		
	T/T	8	2		
	C-allele	107	100		
	T-allele	57	32		
*SLC22A4* rs272893	T/T	34	45	**0.47 (0.2–0.8)** 0.005*	0.8
	T/C	37	11		
	C/C	11	8		
	T-allele	105	101		
	C-allele	59	27		
*SLC22A4* rs272879	G/G	43	32	1.06 (0.6–1.7) 0.8	0.1
	G/C	29	24		
	C/C	10	8		
	G-allele	115	88		
	C-allele	49	40		

*p-value< 0.05, depicted in bold was considered statistically significant

### ASE using TaqMan assay

The log-transformed ratio of VIC (mutant allele) and FAM (wild allele) was used for comparing ASE in responder and non-responder samples for *ACACB* rs2075262, *MTOR* rs1135172, *MTOR* rs1057079, *RPTOR* rs2289764, *SLC22A4* rs272879, *SLC22A4* rs1050152 and *SLC22A4* rs272893 (Table 3). Although the ratio of alleles was skewed towards wild for all the SNPs, there was no significant difference observed in the allelic ratios between responders and non-responders samples.

**Table 3:** Log transformed allelic ratios for selected SNPs in responders and non-responder samples

SNP ID	Allele ratio (log transformed (VIC/FAM)		Mean difference	95% CI interval	*p*-value
	Responders	Non-responders			
rs2075262	− 1.19 ± 0.12	− 1.24 ± 0.15	0.04	− 0.12 to 0.21	0.9
rs1135172	− 1.45 ± 0.17	− 1.39 ± 0.15	− 0.061	− 0.26 to 0.14	0.9
rs1057079	− 1.33 ± 0.17	− 1.45 ± 0.22	0.12	− 0.032 to 0.27	0.9
rs2289764	− 1.41 ± 0.13	− 1.32 ± 0.15	− 0.089	− 0.31 to 0.13	0.3
rs272879	− 1.70 ± 0.14	− 1.74 ± 0.15	0.039	− 0.13 to 0.21	0.98
rs1050152	− 0.81 ± 0.09	− 0.90 ± 0.12	0.09	− 0.13 to 0.31	> 0.9
rs272893	− 1.11 ± 0.11	− 1.15 ± 0.16	0.044	− 0.12 to 0.20	0.9

## Discussion

The field of pharmacogenomics offers a fascinating prospect for improvement of patient care via optimization of the choice and dose of the medicine, reduction of adverse event risk, and further implementation of the principles of personalized medicine. In the case of T2DM, the results of the recent pharmacogenomics have been hurdled by various factors. In the present study, we describe the heterogeneity in response to metformin drug within T2DM individuals. The genetic and transcriptome profiles of drug naïve T2DM patients and healthy controls were assessed to identify variations and DGEs in non-responsive and responsive patients. Our analysis revealed *SLC22A4* gene to be associated with improved response to the metformin drug. The *SLC22A4* is involved in the transport of metformin and oral absorption in the intestine. The presence of variations may lead to lower clearance of the drug. This is in accordance with the previous report of mutant *SLC22A4* mice showing high plasma metformin concentration [39]. The SNPs, rs272893, and rs1050152, were present in disproportion in responders versus non-responders and were selected for validation in a larger cohort. The copy number loss was observed in *PPARGC1A* gene in the non-responders samples. *PPARGC1A* modulates the gluconeogenic targets such as *PCK1* and *G6PC* [40]. The loss in the copy number of the gene may lead to altered gluconeogenesis in the non-responder individuals.

Our analysis of the gene expression led to the identification of four differential expressed gene sets that were able to differentiate non-responders from responders and T2DM patients from healthy controls. Furthermore, these DGEs were found to be involved in key pathways and processes. The overall results highlight the complex heterogeneity among drug naïve T2DM patients before the administration of metformin drug and have the potential for consideration in the early prediction of disease state and response phenotypes.

The overall validity of existing biomarkers for the prediction of metformin drug is still unclear. Metformin is hydrophilic and has to rely on members of the OCT family for entry inside hepatocytes. *OCT1* has been specifically shown to be associated with the therapeutic efficacy of metformin [41]. The targets of metformin include mitochondrial complex I and AMPK, which regulate cellular energy homeostasis [42]. The studies to predict metformin drug response have relied on the existing target and treatment on a cell line or mouse models. Udhane et al. [29] have shown novel links between metformin response and energy metabolism, sex steroid biosynthesis, the cell cycle, and the immune system in H295R cells. Recently, Park et al. have identified metabolites, namely citric acid, myoinositol, and hippuric acid, as markers of metformin response in early-phase T2DM patients [28]. However, we followed the clinical phenotypes' transcriptome patterns, i.e., metformin response before the drug administration, to predict early signatures for drug response predictions. We identified the *IDO2* gene as highly upregulated in non-responders compared with responders and healthy controls with a fold change of 6.3 and 4.1, respectively. *IDO2* gene plays an essential role in tryptophan amino acid catabolism and leads 95% of the amino acid to the kynurenine pathway [43]. The metabolites of the kynurenine pathway are reported to be elevated in individuals with insulin resistance before the manifestation of hyperglycemia [44].

Moreover, Muzik et al. showed the stabilization of tryptophan metabolism by decreasing contribution from the kynurenine metabolic pathway upon metformin treatment [44]. Higher levels of the *IDO2* gene in early T2DM subjects may indicate response features of future metformin therapy. Besides the *IDO2* gene, *FRMD6*, *DMGDH*, and *NMNAT3* genes were also highly upregulated in non-responders and are associated with glycine, serine, and threonine metabolism, hippo signalling, and nicotinate and nicotinamide metabolism, respectively. The role of metformin as an inhibitor of the transactivation of YAP, which is a key effector of the Hippo pathway, is reported [45]. On the contrary, *FRMD6* gene overexpression has been shown to activate Hippo signalling and induce translocation of YAP from the nucleus to the cytoplasm [46]. *DMGDH* gene is known to catalyze the conversion of glycine to sarcosine, and hypoglycemia is reported as a risk for developing diabetes [47]. T2DM adults metabolize nicotine more rapidly [48], *NMNAT3* genes overexpression in our data may provide a possible explanation for the nicotine addiction of T2DM, as highlighted before [48]. *HSD11B1* gene was another gene that was found upregulated four-fold higher in responders and downregulated four-fold lower in non-responders. Although the expression is higher in abdominal adipose tissue, many genetic abnormalities have been implicated in abnormal glucose metabolism, risk of diabetes, and body fat distribution [49, 50].

The correlation of response to metformin therapy with gene expression highlighted *GDF15*, *TWISTNB,* and *RPL36A* genes. GDF15 is a cytokine from the TGF-β superfamily associated with hyperglycemia and risk of incident diabetes [51]. *TWISTNB* gene expression has been linked to the risk of Uterine Leiomyoma [52] and early-stage laryngeal cancers [53]. *RPL36A* gene is involved in tumour cell proliferation and associated with gliomas [54, 55]. We showed that *GDF15,* *TWISTNB* and *RPL36A* gene expression in drug naïve T2DM individuals could be used to predict the response to metformin therapy. The DGEs presented here provide early targets in newly diagnosed T2DM for metformin drug response prediction.

The prediction or prognosis of T2DM, despite noteworthy advancements in genome-wide association studies, is still weak. Previous evaluation of T2DM transcriptomes has highlighted *TAF1* and *MAFK* as potential target genes for impaired fasting glucose and T2DM [56]. *CDK5*, *CDKN2A*, *THADA*, and *CAPN10* expression levels were also highlighted in a blood-based analysis of T2DM susceptibility genes [57]. However, our study highlighted *CXXC4, GDF2, MID1,* and *NDUFA4L2* genes to be highly expressed in both responders and non-responders compared to healthy controls. Also, *IDO1, LAMP3*, and *CXCL10* genes are poorly expressed in both responders and non-responders compared to healthy controls. These genes are involved in Wnt signalling, cytokine–cytokine receptor interaction, ubiquitin-mediated proteolysis, oxidative phosphorylation, tryptophan metabolism, and adaptive immunity. Furthermore, our disease gene association analysis highlighted rs113805659 in the *MAGI2* gene in relation to diabetes in African Americans [38]. All these pathways and genes have been previously implicated in the risk of T2DM and its complications.

The ASE refers to the preferential expression of one of the two alleles in a hybrid under the influence of regulatory elements or sequences from the genome. We evaluated ASEs for SNPs selected from our exome and transcriptome date. However, the constraints in TaqMan probe design limited our analysis to few SNPs among which rs272893 and rs1050152 were found to be associated with better metformin response. None of the SNPs showed a statistical significance. Interestingly it was observed that the expression of the alleles was skewed towards the wild allele in the majority of the samples evaluated.

## Conclusions

The present study provides a valuable resource for the genetic variations and transcriptome profile of drug naïve T2DM individuals. The identification of variations in the *SLC22A4* gene holds promise for drug response prediction but needs further evaluation in larger cohorts. The expression markers generated from the transcriptome profile can be further evaluated for their role in T2DM predisposition and metformin drug response.

## Supplementary Information

Below is the link to the electronic supplementary material.

Supplementary file1 (DOCX 21 KB)

Supplementary Fig. 1. RNA-Seq data normalization (a) Density of logcpm values for raw reads, (b) Density of logcpm values for normalised reads, (c) Logcpm for individual samples before data normalization and (d) Logcpm for individual samples after data normalization (PDF 196 KB)

Supplementary Fig. 2. RNA-Seq differential expression (a) Multidimensional scaling (MDS) plot showing distribution of control, non-responders and responders samples, (b) Volcano plot showing log fold change (logFC) on the x-axis and -log10 (P-value) on the y-axis of gene expression alterations found using edgeR. Genes with FDR > 0.05 red dots (PDF 122 KB)

Supplementary Fig. 3. Differentially expressed genes in control, non-responders and responders samples (a) Upregulated and (b) Downregulated (PDF 69 KB)

Supplementary Fig. 4. Gene Ontology (GO) analysis of genes in Metformin_Set_1. (a) Biological process, (b) Molecular function, (c) Cellular component and (d) Enrichment for GO groups (PDF 490 KB)

Supplementary Fig. 5. Gene Ontology (GO) analysis of genes in T2D_Set_1 (a) Biological process, (b) Molecular function, (c) Cellular component and (d) Enrichment for GO groups (PDF 500 KB)

Supplementary Fig. 6. Gene Ontology (GO) analysis of genes in T2D_Set_2. (a) Biological process, (b) Molecular function, (c) Cellular component and (d) Enrichment for GO groups (PDF 475 KB)

Supplementary Fig. 7. Gene Ontology (GO) analysis of genes in Metformin_Set_2. (a) Biological process, (b) Molecular function, (c) Cellular component and (d) Enrichment for GO groups (PDF 394 KB)

Supplementary Fig. 8. Association of gene expression with change in HbA1c levels after 3 months of metformin therapy (a) Correlation between gene expression and change in HbA1c levels; (b) Change in HbA1c against GDF15; (c) Change in HbA1c against TWISTNB and (d) Change in HbA1c against RPL36A. (NRC, normalized read count) (PDF 798 KB)

Supplementary Fig. 9. Identification of variants associated with different diseases in enriched genes (PDF 222 KB)

Supplementary file11 (DOCX 58 KB)

## Data Availability

The datasets used and analyzed during the current study are available from the corresponding author on reasonable request.
