# Supplementary material for: Implications of genetic variations, differential gene expression, and allele-specific expression on metformin response in drug-naïve type 2 diabetes
Source: J Endocrinol Invest. 2022 Dec 18;46(6):1205–18. doi: 10.1007/s40618-022-01989-y (PMC10185588; doi:10.1007/s40618-022-01989-y)
Supplement: Supplementary file 1 — Supplementary file1 (DOCX 21 KB) [file 40618_2022_1989_MOESM1_ESM.docx]

Supplemental Information

**Implications of genetic variations, differential gene expression, and allele-specific expression on metformin response in drug-naïve type 2 diabetes**

Manik Vohra^1^, Anu Radha Sharma^1^, Sandeep Mallya^2^, Navya B Prabhu^1^, Pradyumna Jayaram^3^, Shivashankar K Nagri^4^, Shashikiran Umakanth^5^, Padmalatha S Rai^1*^

^1^Department of Biotechnology, Manipal School of Life Sciences, Manipal Academy of Higher Education, Manipal, India

^2^Department of Bioinformatics, Manipal School of Life Sciences, Manipal Academy of Higher Education, Manipal, India

^3^Department of Cell and Molecular Biology, Manipal School of Life Sciences, Manipal Academy of Higher Education, Manipal, India

^4^Department of Medicine, Kasturba Medical College, Manipal Academy of Higher Education, Manipal, India

^5^Department of Medicine, Dr. T.M.A. Pai Hospital, Manipal Academy of Higher Education, Manipal, India

***Corresponding author:**

Email: padmalatha.rai@manipal.edu

Orcid id: 0000-0001-7159-0560

**Study subjects**

The participants with malignancies, endocrine disorders, renal or liver complications, pregnant or lactating women and who are under corticosteroid treatment were excluded from the study. We included T2DM participants undergoing metformin as monotherapy after the diagnosis of the disease. A complete record of the family history, medication adherence, details of any earlier complications, smoking or alcohol habits was compiled. The glycaemic index of the T2DM participants was recorded at the time of enrolment and post metformin monotherapy of 3 months. The response to metformin was defined as the decrease in glycated haemoglobin (HbA1c) level >1% or >20mg/dL decrease in fasting blood glucose level from baseline after three months of therapy.

**Targeted exome sequencing**

The list of genes (n=22) involved in metformin pharmacodynamics and pharmacokinetics pathway was formulated and used for the target panel design. The details of the selected genes are presented in Supplementary Table 1. The designed panel captured 97.73% of the exonic region of 22 genes.

The DNA was isolated from freshly collected blood samples of responders (n=13), non-responders (n=17) and healthy controls (n=15) and quantified using Qubit dsDNA HS Assay Kit (ThermoFisher Scientific, US). The quantified DNA samples were used for library preparation with Ion AmpliSeq DNA library kit (ThermoFisher Scientific, US) and primer pools of designed target panel according to manufacturer’s protocols (ThermoFisher Scientific, US). Each library was labelled with different barcodes using Ion Express Barcode Adapter kit. Using chip calculator tool in Ion AmpliSeq Designer, we calculated the number of libraries for pooling based on 30X coverage of 1068 amplicons per library from designed panel. The pooled samples were subjected to emulsion PCR and enrichment using Ion PGM Template OT2 kit using manufacturer’s instruction. The enriched ion sphere particles were loaded on PGM 318 chip and sequencing was carried on Ion PGM system.

**Bioinformatic analysis and variant calling**

The raw reads obtained from sequencing were aligned with reference human sequence (human genome build hg19). The variant calling was performed on Ion Torrent Variant Caller v5.0 in Torrent Suite software v5.0 (ThermoFisher Scientific). The low quality and noisy reads were removed and using Ion Torrent Variant Caller Plugin sequence variants were predicted. The vcf files generated were transferred to Ion Reporter software (ThermoFisher Scientific, US). In the Ion reporter software, we used predefined workflow for annotating variants for each sample file. The annotated variants from each sample were further grouped based on responder, non-responder and healthy control status, and analysed using Maftools.

**Copy number analysis**

To estimate the copy number alterations from targeted exome data CNVKit (v0.9.2) was used with default settings. The expected read counts were generated for each genomic window from a set of normal samples and the log-ratio of the read counts from the responders (n=13) and non-responder (n=17) samples relative to normal samples was calculated. We used 15 normal samples to generate the normal read count for each genomic window. Using CNVit’s batch pipeline, we created reference value and calculated the log_2_ ratio, segmentation of those log-ratios, and estimated the integral CNV in responders and non-responder samples. The segments with an integral copy of one or less were considered as deletions and copy number with three or more were considered as duplications.

**Whole transcriptome sequencing**

1 µg of total RNA was subjected to mRNA enrichment using dynabeads oligo (dT)_25_ (Thermo Fisher Scientific, Inc., US), and the eluted mRNA was used for library construction and sequencing. Ion total RNA-seq kit v2 (Thermo Fisher Scientific, Inc., US) was used to construct whole transcriptome libraries from 1-500 ng of enriched poly(A) RNA according to manufacturer’s protocols. Agencourt Ampure XP beads (Beckman Coulter, Inc., Brea, CA, USA) were used for all clean-up steps. After barcoding, amplification, and purification of the library, an Agilent 2100 Bioanalyzer was used to check the quality and quantify the library. The sequencing of RNA libraries was performed on P1 v3 chips using Ion Proton System (Thermo Fisher Scientific, Inc., USA). The transcriptome data was deposited at NCBI (accession no. GEO: GSE153315).

**Data processing for transcriptome analysis**

Transcriptome sequencing data was analysed on Torrent Suite (Version 5.10.0) using the RNASeq Analysis plugin (5.2.0.5). The two-step alignment technique was followed for mapping sequencing reads to the human genome. Firstly, the STAR aligner was used to map reads to the hg19 version of the human genome. Secondly, the unmapped reads in the first step were aligned using bowtie2. Mapped reads from both the alignments were merged with Picard tools to obtain the read counts. The high-quality aligned reads were generated from the filtered data and saved as ‘bam’ files. These combined aligned files were used for all subsequent analyses.

**Differential gene expression analysis**

Differential expression analysis was performed using the edgeR (3.14.0), a bioconductor package. The genes with read count <10 in more than 75% of the samples were excluded in the first step. The read counts were transformed into counts per million (cpm) and genes with cpm > 0.5 in at least 2 libraries were selected for normalization. The differential expression of each gene was determined by the comparison between non-responders, responders, and healthy control groups, and fold change (FC) was calculated. Differentially expressed genes were selected on the criteria i.e., FC >2 (upregulated) or < -2 (downregulated), FDR <5% and p-value < 0.05.

**Allele specific expression (ASE) analysis**

The ASE of the heterozygous sites in the genome was assessed by using the corresponding transcriptome date. ASEQ v1.1.8 tool was used to compare the genotype information obtained from the targeted exome sequencing data to the transcriptome data. For both targeted exome and transcriptome data, the threshold minimum score of 20 was employed for base quality and read quality, and minimum depth of coverage at that position was set at 10. Finally, Fisher exact test with a p-value of ≤0.01 was employed to control the false positive calls arising due to depth of coverage between the targeted exome and transcriptome data. The ASE from NGS data was defined as the ratio of reads at the SNP sites in transcriptome profile as ≤ 0.45 or ≥ 0.55. The high frequency SNPs which showed ASE were selected for further validation in study subjects.
