# Supplementary figures and images for: Implications of genetic variations, differential gene expression, and allele-specific expression on metformin response in drug-naïve type 2 diabetes
Source: J Endocrinol Invest. 2022 Dec 18;46(6):1205–18. doi: 10.1007/s40618-022-01989-y (PMC10185588; doi:10.1007/s40618-022-01989-y)

**a) Raw data**

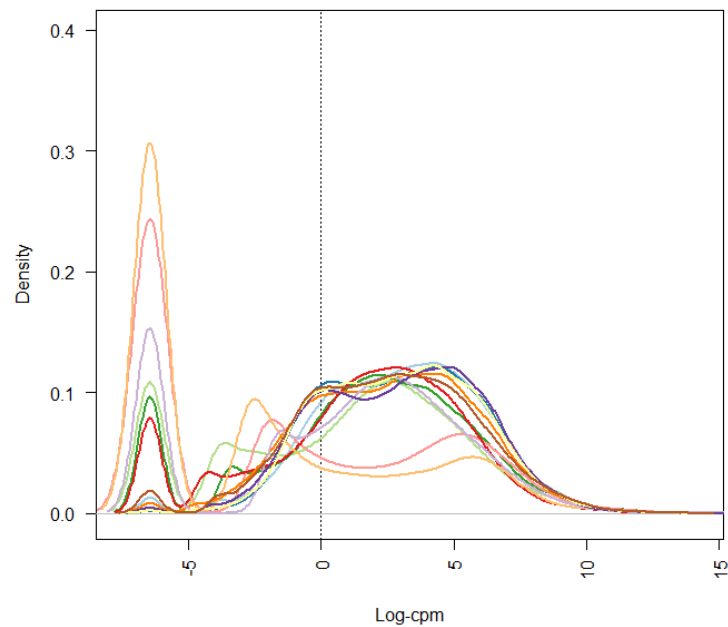

**b) Filtered data**

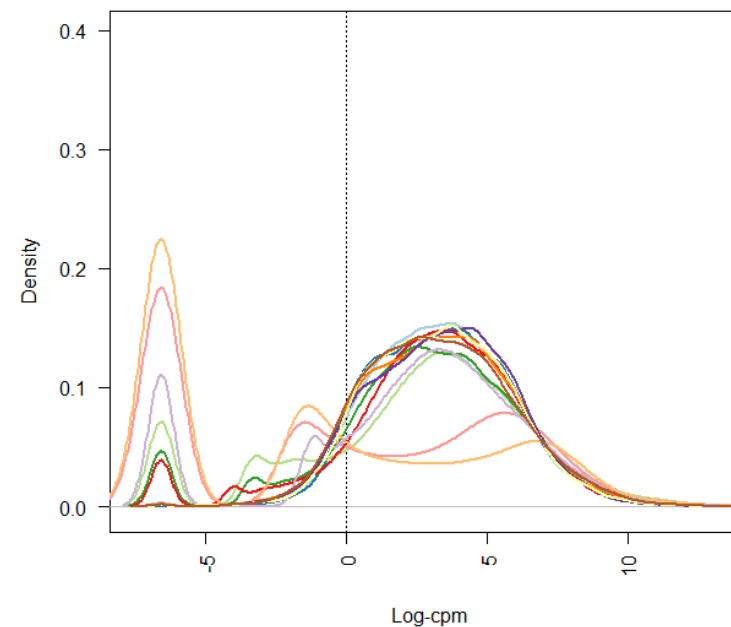

**c) Unnormalised data**

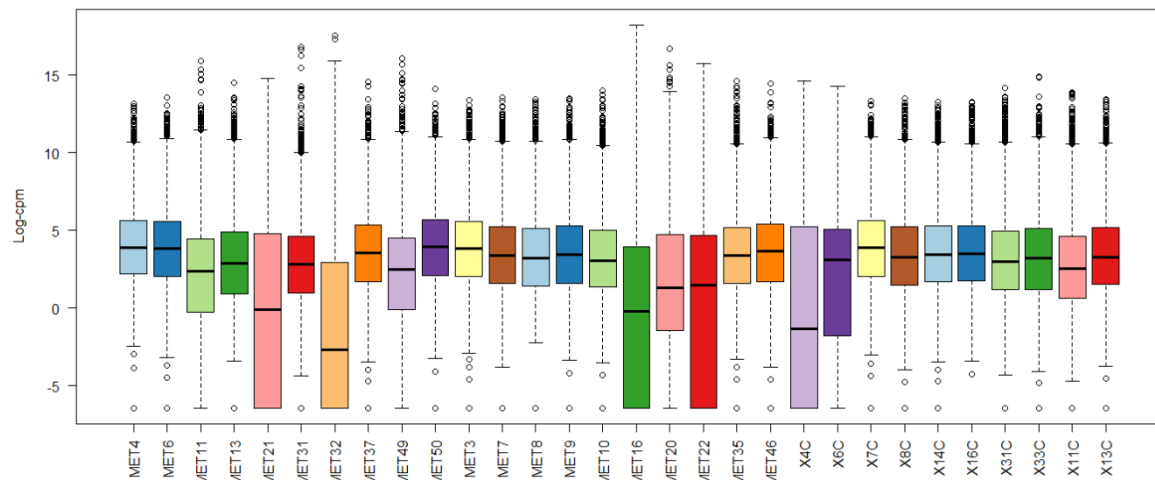

**d) Normalised data**

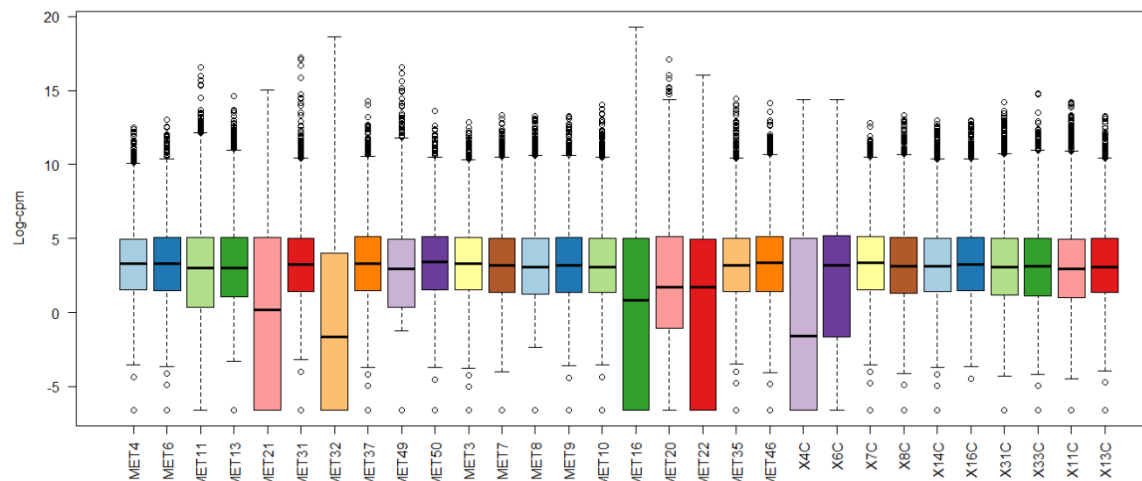

Supplement: Supplementary file 2 — Supplementary Fig. 1. RNA-Seq data normalization (a) Density of logcpm values for raw reads, (b) Density of logcpm values for normalised reads, (c) Logcpm for individual samples before data normalization and (d) Logcpm for individual samples after data normalization (PDF 196 KB) [file 40618_2022_1989_MOESM2_ESM.pdf]

a)

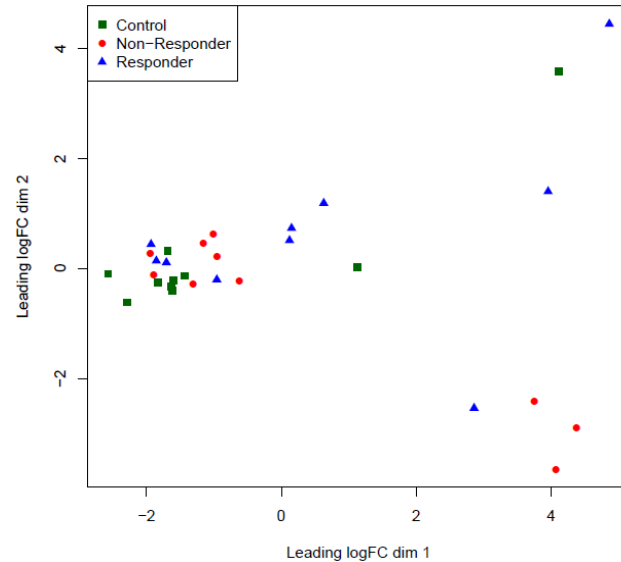

**MDS plot**

b)

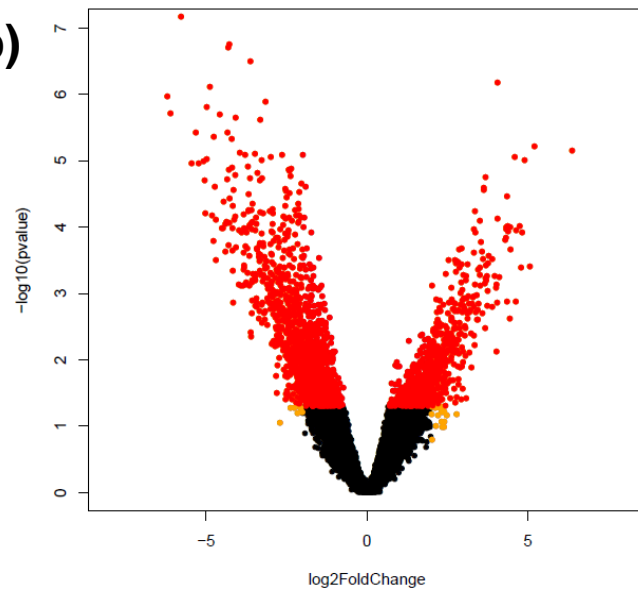

**Non-responder v/s Responder**

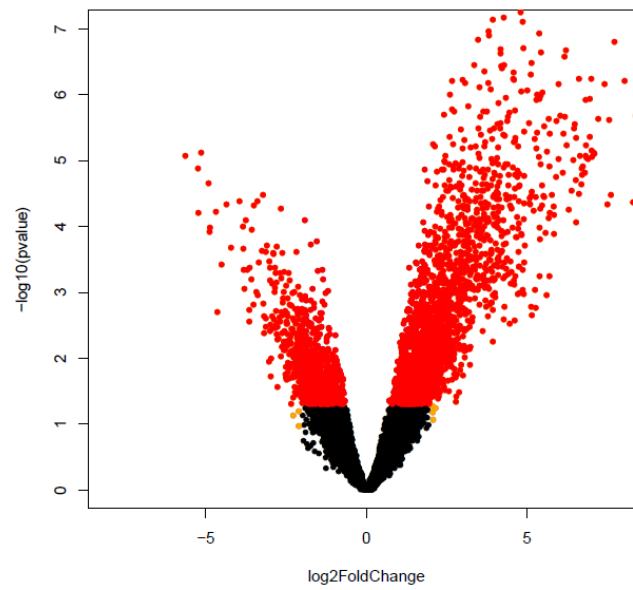

**Responder v/s Control**

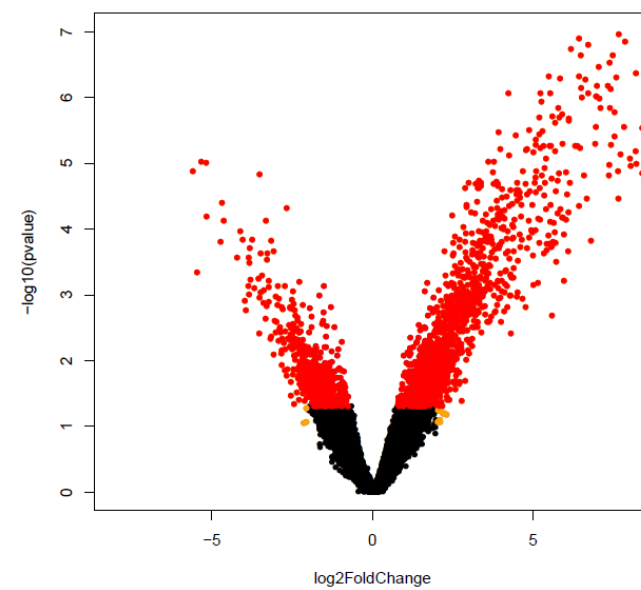

**Non-responder v/s Control**

Supplement: Supplementary file 3 — Supplementary Fig. 2. RNA-Seq differential expression (a) Multidimensional scaling (MDS) plot showing distribution of control, non-responders and responders samples, (b) Volcano plot showing log fold change (logFC) on the x-axis and -log10 (P-value) on the y-axis of gene expression alterations found using edgeR. Genes with FDR > 0.05 red dots (PDF 122 KB) [file 40618_2022_1989_MOESM3_ESM.pdf]

a)

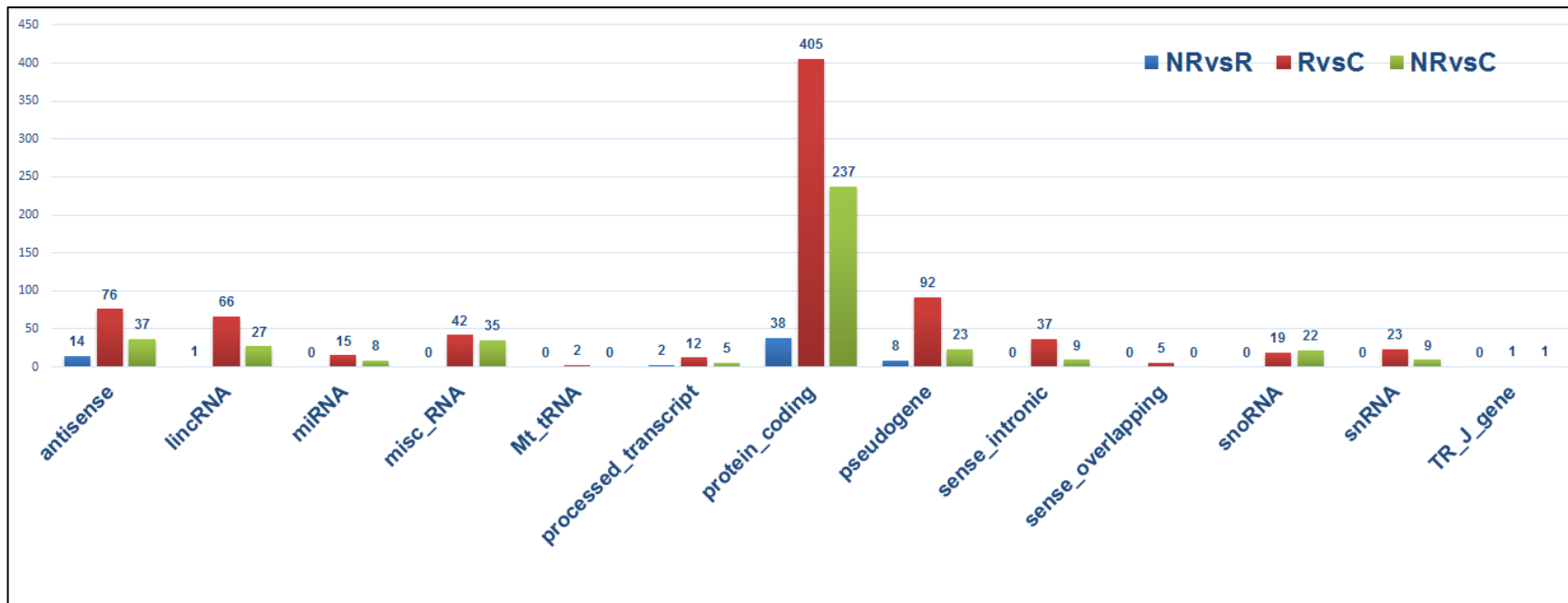

b)

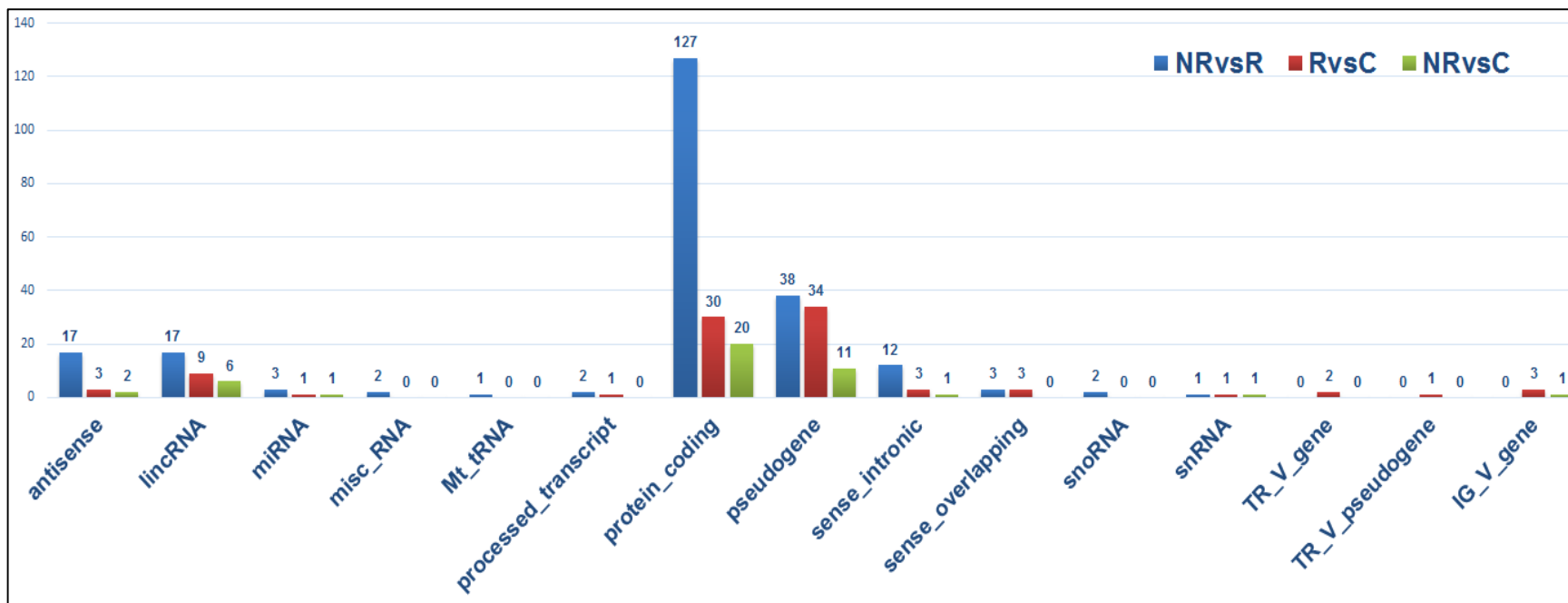

Supplement: Supplementary file 4 — Supplementary Fig. 3. Differentially expressed genes in control, non-responders and responders samples (a) Upregulated and (b) Downregulated (PDF 69 KB) [file 40618_2022_1989_MOESM4_ESM.pdf]

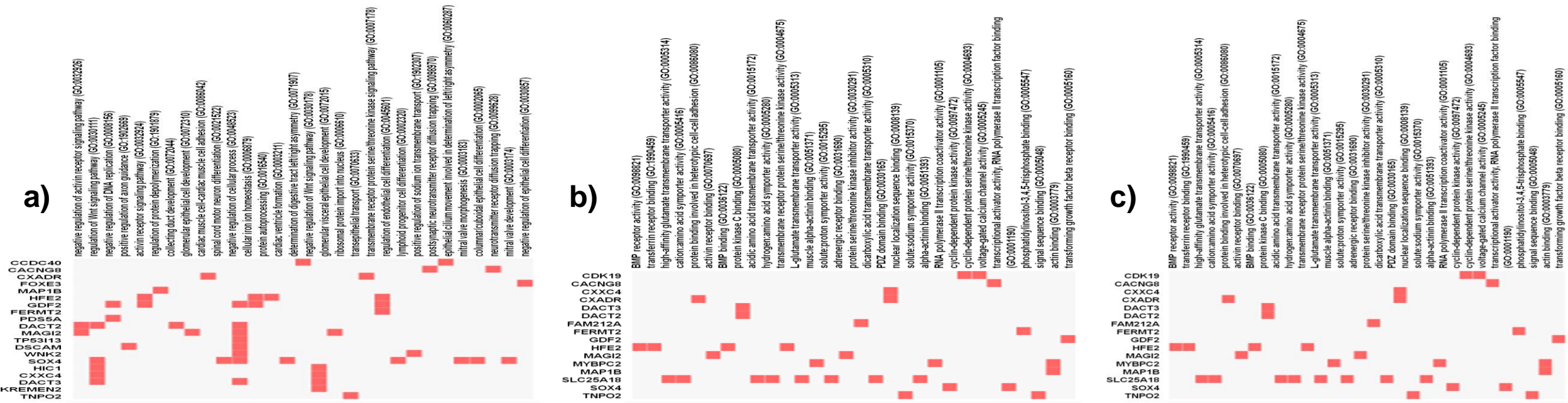

Biological Process

Molecular Function

Cellular Component

d)

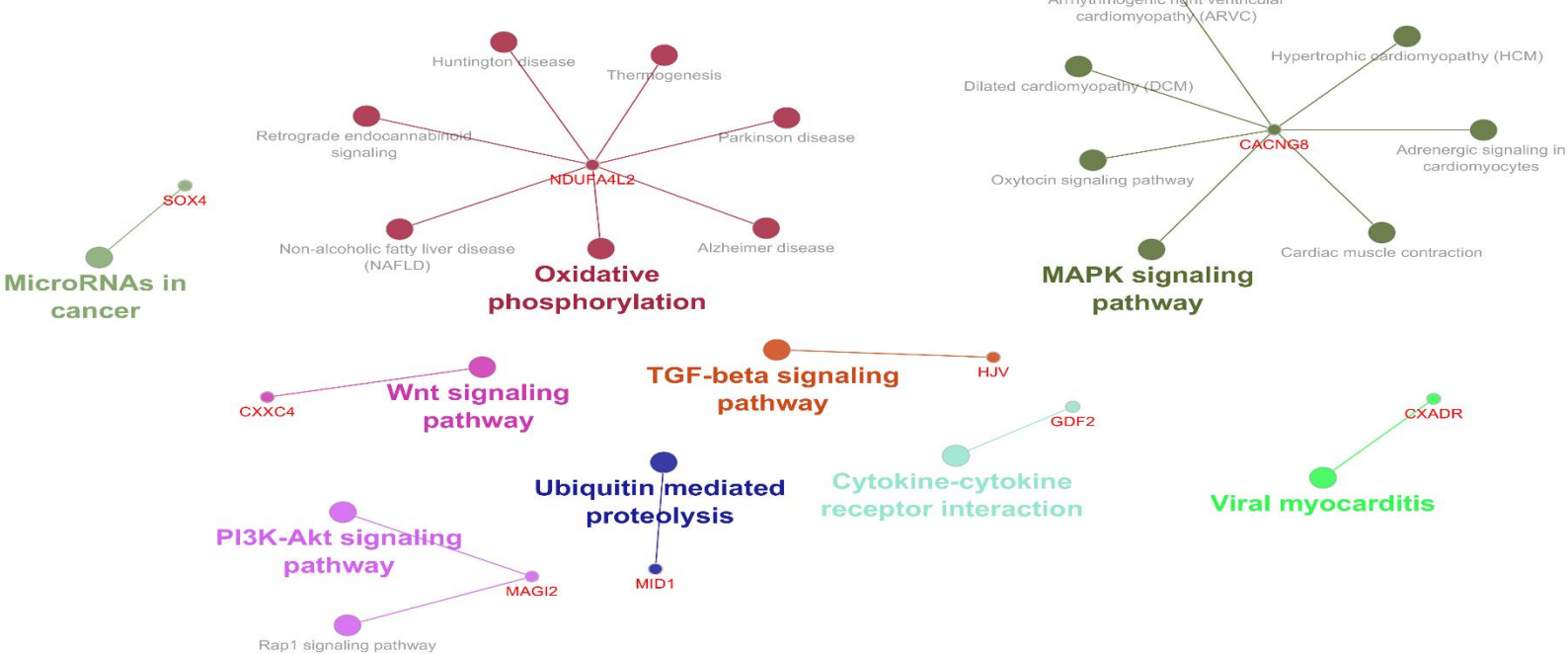

Supplement: Supplementary file 6 — Supplementary Fig. 5. Gene Ontology (GO) analysis of genes in T2D_Set_1 (a) Biological process, (b) Molecular function, (c) Cellular component and (d) Enrichment for GO groups (PDF 500 KB) [file 40618_2022_1989_MOESM6_ESM.pdf]

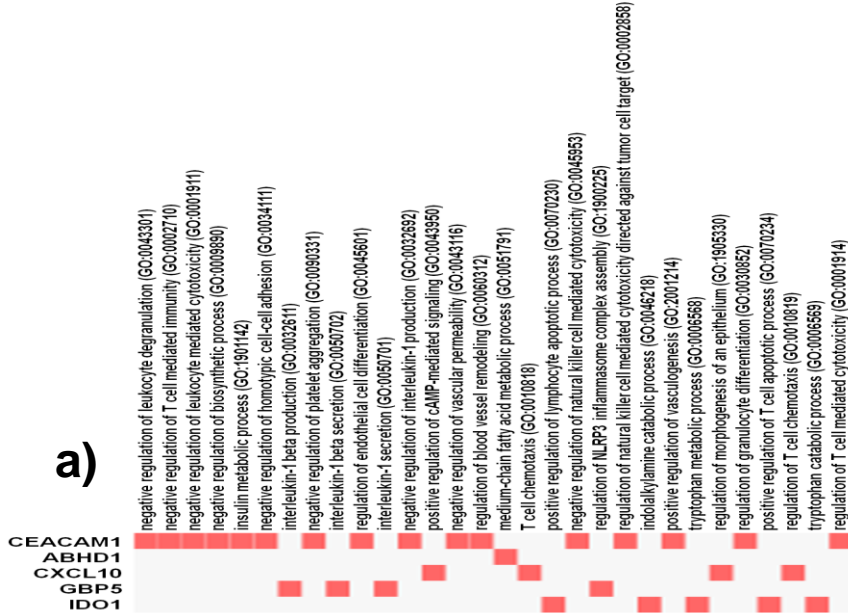

## Biological Process

**b)**

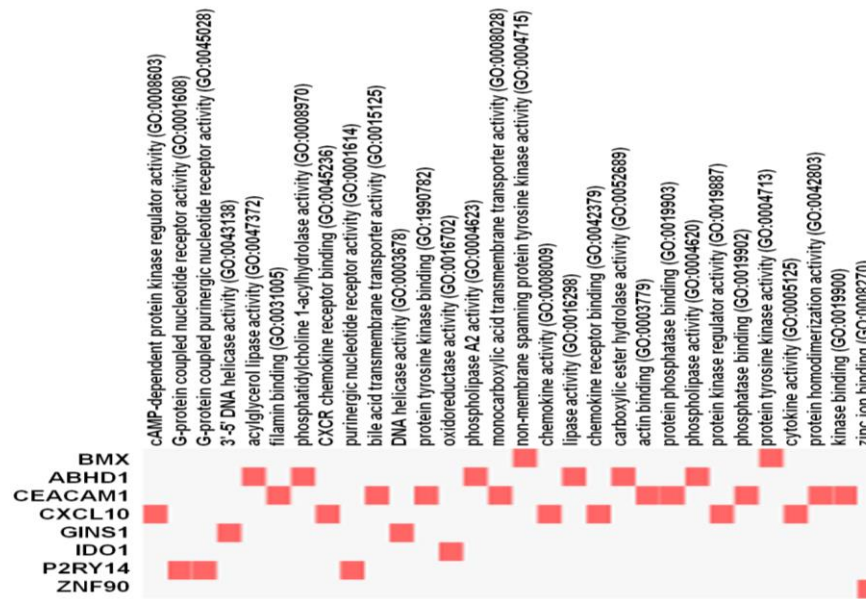

## Molecular Function

**c)**

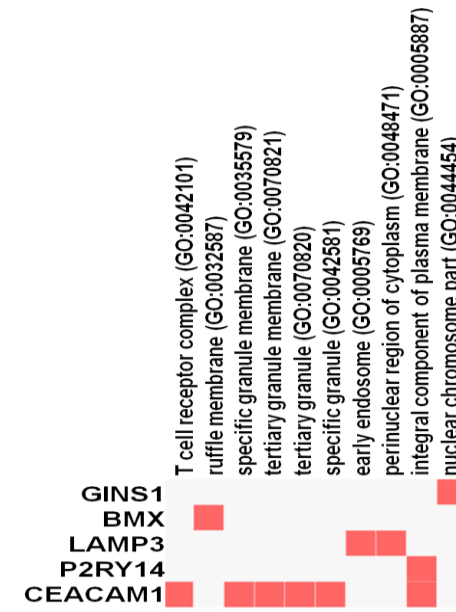

## Cellular Component

**d)**

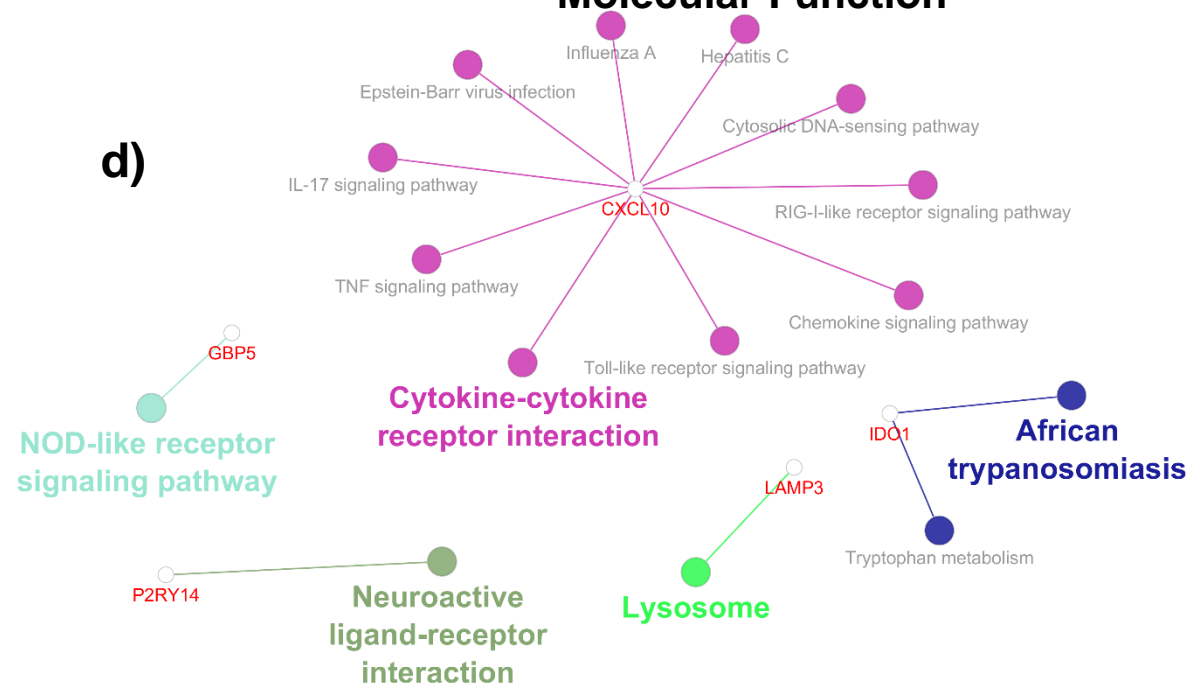

Supplement: Supplementary file 7 — Supplementary Fig. 6. Gene Ontology (GO) analysis of genes in T2D_Set_2. (a) Biological process, (b) Molecular function, (c) Cellular component and (d) Enrichment for GO groups (PDF 475 KB) [file 40618_2022_1989_MOESM7_ESM.pdf]

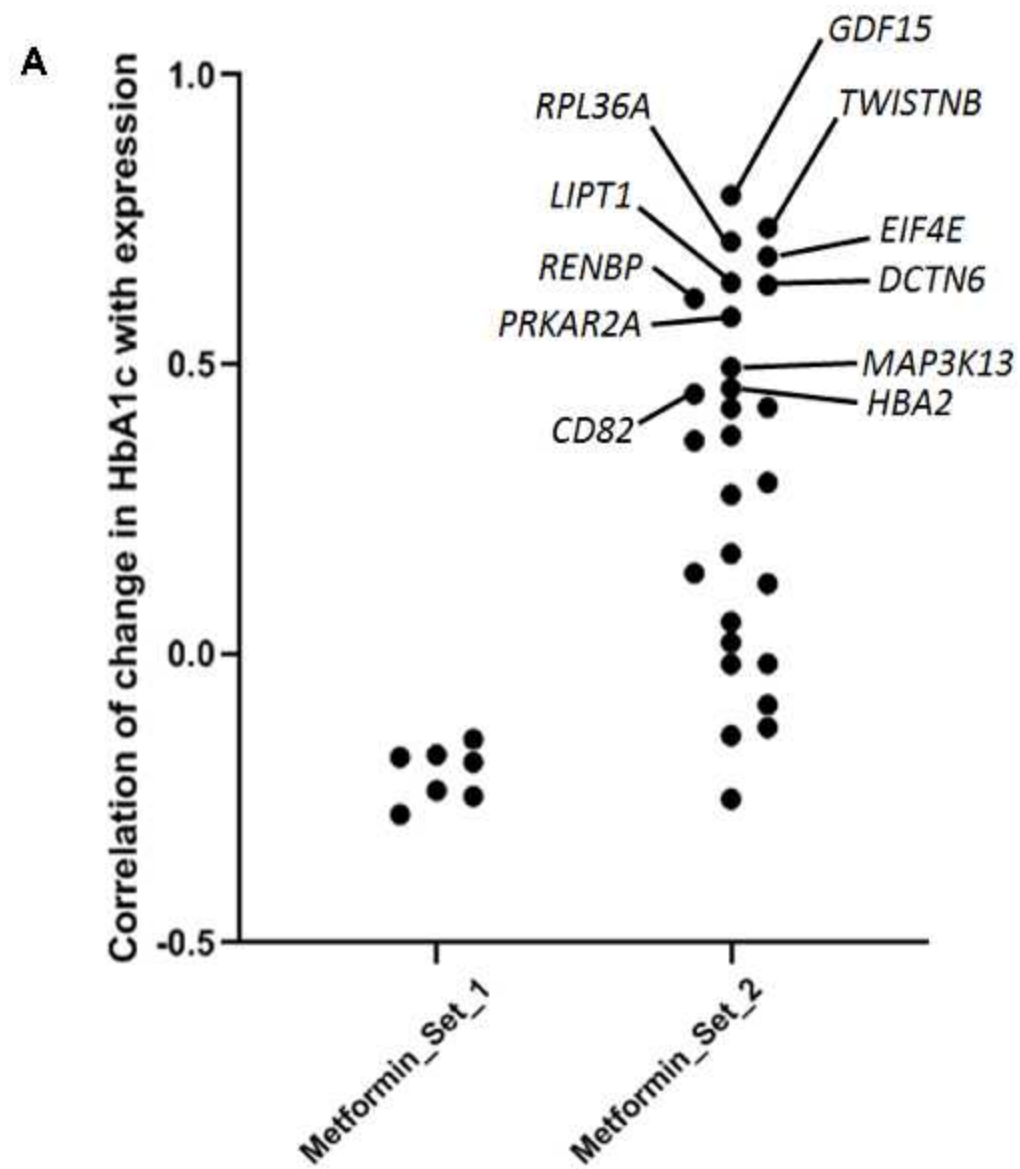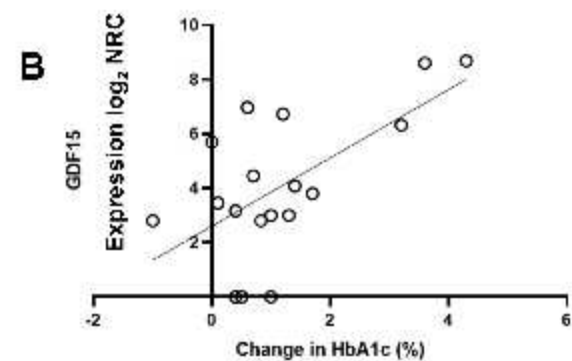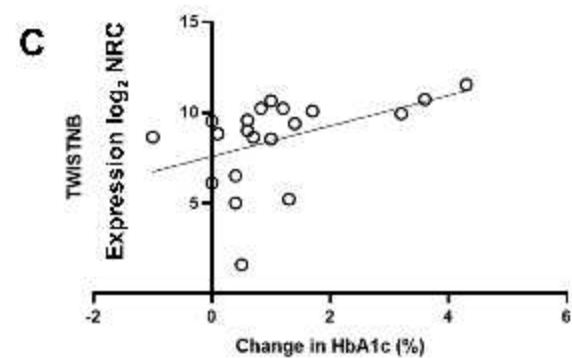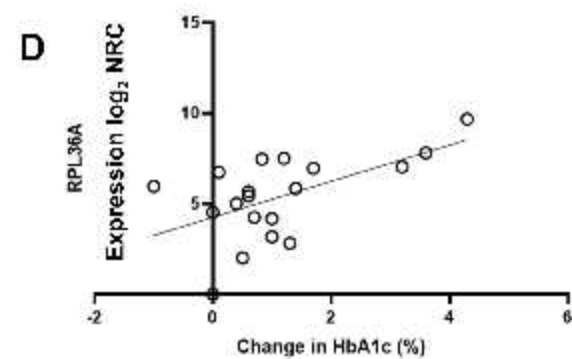

Supplement: Supplementary file 9 — Supplementary Fig. 8. Association of gene expression with change in HbA1c levels after 3 months of metformin therapy (a) Correlation between gene expression and change in HbA1c levels; (b) Change in HbA1c against GDF15; (c) Change in HbA1c against TWISTNB and (d) Change in HbA1c against RPL36A. (NRC, normalized read count) (PDF 798 KB) [file 40618_2022_1989_MOESM9_ESM.pdf]

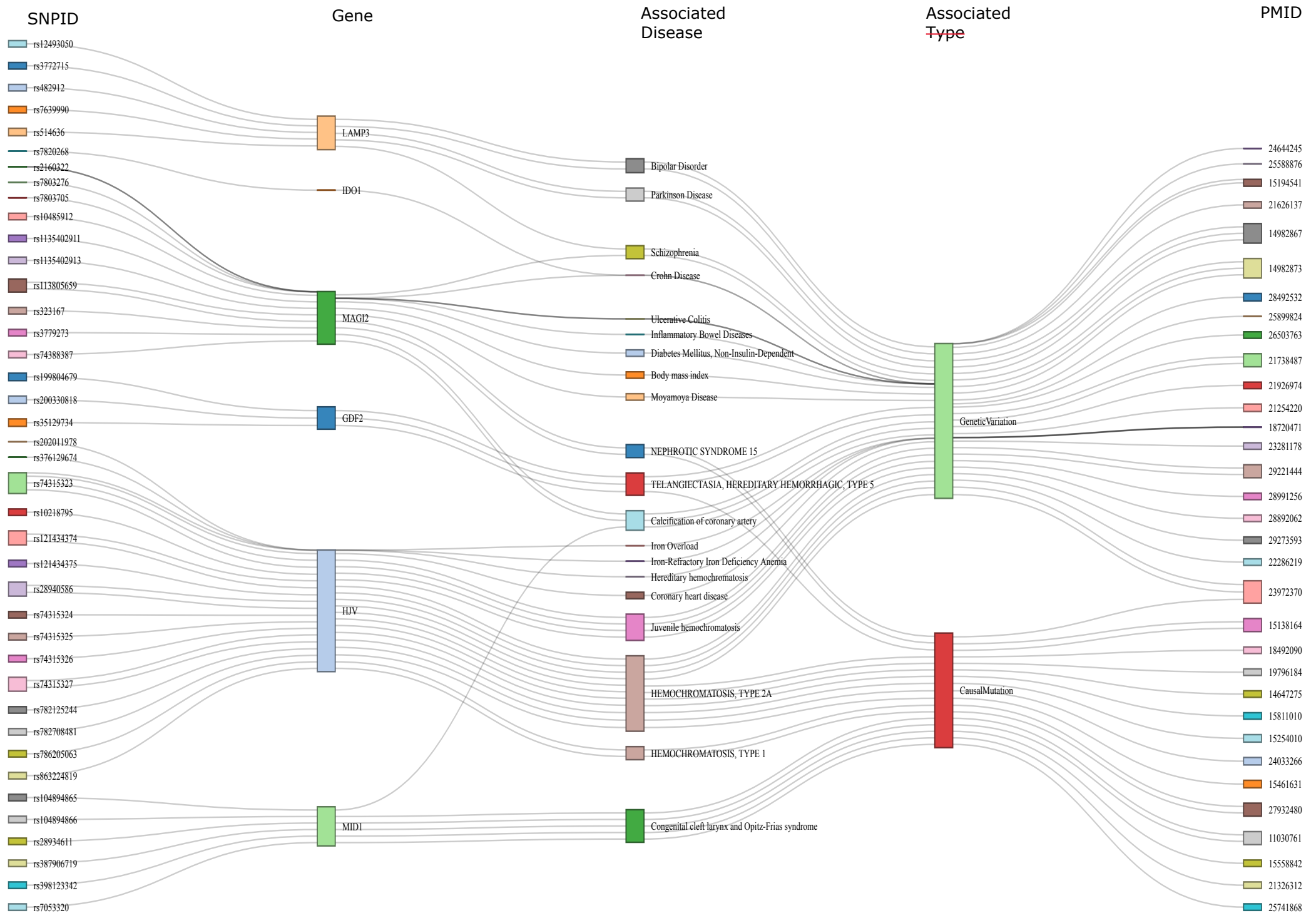

Supplement: Supplementary file 10 — Supplementary Fig. 9. Identification of variants associated with different diseases in enriched genes (PDF 222 KB) [file 40618_2022_1989_MOESM10_ESM.pdf]
