# Supplementary material for: Implications of genetic variations, differential gene expression, and allele-specific expression on metformin response in drug-naïve type 2 diabetes
Source: J Endocrinol Invest. 2022 Dec 18;46(6):1205–18. doi: 10.1007/s40618-022-01989-y (PMC10185588; doi:10.1007/s40618-022-01989-y)
Supplement: Supplementary file 5 — Supplementary Fig. 4. Gene Ontology (GO) analysis of genes in Metformin_Set_1. (a) Biological process, (b) Molecular function, (c) Cellular component and (d) Enrichment for GO groups (PDF 490 KB) [file 40618_2022_1989_MOESM5_ESM.pdf]

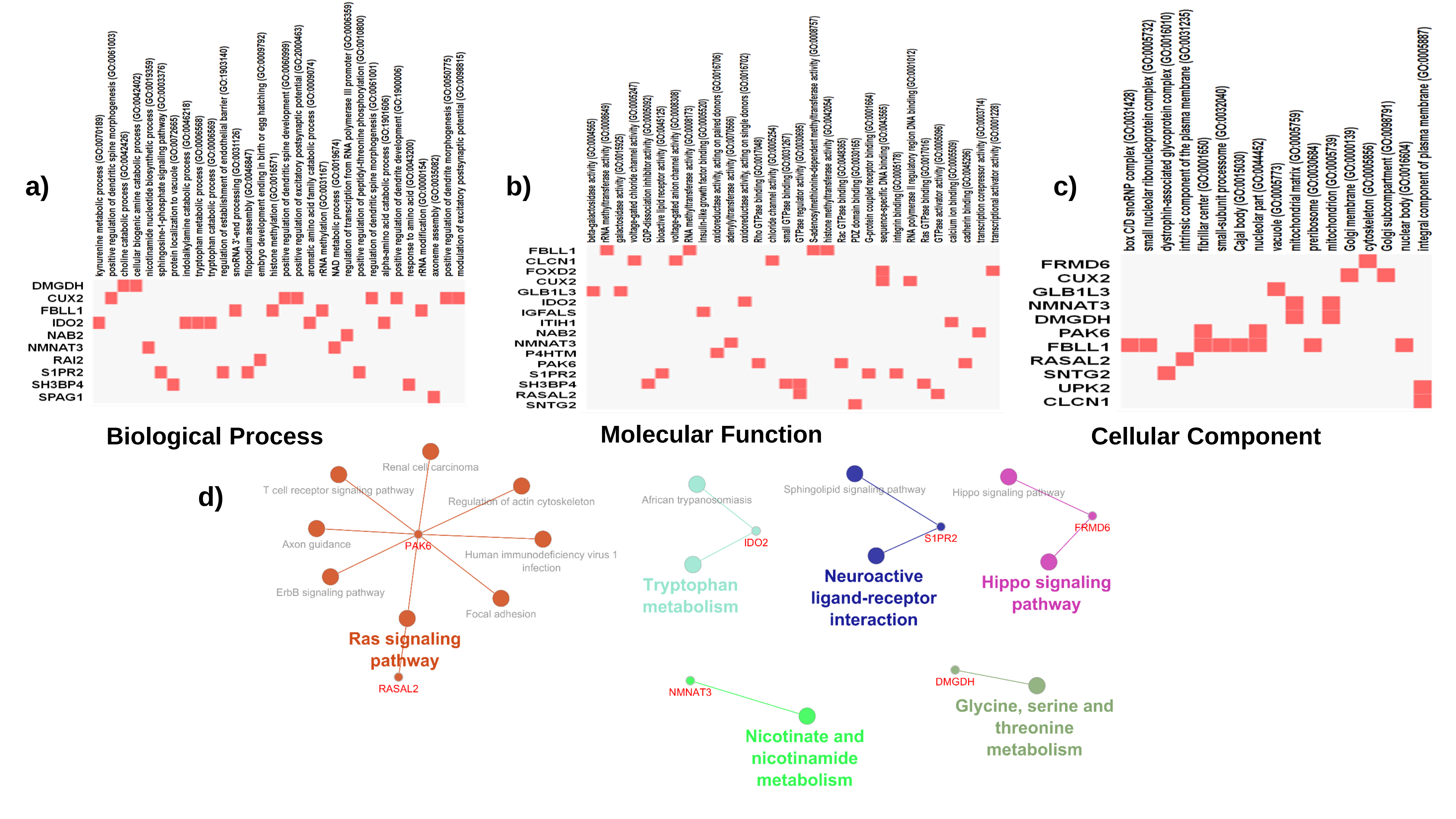
a)
kynurenine metabolic process (GO:0070189)
positive regulation of dendritic spine morphogenesis (GO:0061003)
choline catabolic process (GO:0042426)
cellular biogenic amine catabolic process (GO:0042402)
nicotinamide nucleotide biosynthetic process (GO:0019359)
sphingosine-1-phosphate signaling pathway (GO:0003376)
protein localization to vacuole (GO:0072665)
indolalkylamine catabolic process (GO:0046218)
tryptophan metabolic process (GO:0006568)
tryptophan catabolic process (GO:0006569)
regulation of establishment of endothelial barrier (GO:1903140)
snoRNA 3'-end processing (GO:0031126)
filopodium assembly (GO:0046847)
embryo development ending in birth or egg hatching (GO:0009792)
histone methylation (GO:0016571)
positive regulation of dendritic spine development (GO:0060999)
positive regulation of excitatory postsynaptic potential (GO:2000463)
aromatic amino acid family catabolic process (GO:0009074)
rRNA methylation (GO:0031167)
NAD metabolic process (GO:0019674)
regulation of transcription from RNA polymerase III promoter (GO:0006359)
positive regulation of peptidyl-threonine phosphorylation (GO:0010800)
regulation of dendritic spine morphogenesis (GO:0061001)
alpha-amino acid catabolic process (GO:1901606)
positive regulation of dendrite development (GO:1900006)
response to amino acid (GO:0043200)
rRNA modification (GO:0000154)
axoneme assembly (GO:0035082)
positive regulation of dendrite morphogenesis (GO:0050775)
modulation of excitatory postsynaptic potential (GO:0098815)
DMGDH
CUX2
FBLL1
IDO2
NAB2
NMNAT3
RAI2
S1PR2
SH3BP4
SPAG1
Biological Process
b)
beta-galactosidase activity (GO:0004565)
rRNA methyltransferase activity (GO:0008649)
galactosidase activity (GO:0015925)
voltage-gated chloride channel activity (GO:0005247)
GDP-dissociation inhibitor activity (GO:0005092)
bioactive lipid receptor activity (GO:0045125)
voltage-gated anion channel activity (GO:0008308)
RNA methyltransferase activity (GO:0008173)
insulin-like growth factor binding (GO:0005520)
oxidoreductase activity, acting on paired donors (GO:0016706)
adenylyltransferase activity (GO:0070566)
oxidoreductase activity, acting on single donors (GO:0016702)
Rho GTPase binding (GO:0017048)
chloride channel activity (GO:0005254)
small GTPase binding (GO:0031267)
GTPase regulator activity (GO:0030695)
S-adenosylmethionine-dependent methyltransferase activity (GO:0008757)
histone methyltransferase activity (GO:0042054)
Rac GTPase binding (GO:0048365)
PDZ domain binding (GO:0030165)
G-protein coupled receptor binding (GO:0001664)
sequence-specific DNA binding (GO:0043565)
integrin binding (GO:0005178)
RNA polymerase II regulatory region DNA binding (GO:0001012)
Ras GTPase binding (GO:0017016)
GTPase activator activity (GO:0005096)
calcium ion binding (GO:0005509)
cadherin binding (GO:0045296)
transcription corepressor activity (GO:0003714)
transcriptional activator activity (GO:0001228)
FBLL1
CLCN1
FOXD2
CUX2
GLB1L3
IDO2
IGFALS
ITIH1
NAB2
NMNAT3
P4HTM
PAK6
S1PR2
SH3BP4
RASAL2
SNTG2
Molecular Function
c)
box C/D snoRNP complex (GO:0031428)
small nucleolar ribonucleoprotein complex (GO:0005732)
dystrophin-associated glycoprotein complex (GO:0016010)
intrinsic component of the plasma membrane (GO:0031235)
fibrillar center (GO:0001650)
small-subunit processome (GO:0032040)
Cajal body (GO:0015030)
nucleolar part (GO:0044452)
vacuole (GO:0005773)
mitochondrial matrix (GO:0005759)
preribosome (GO:0030684)
mitochondrion (GO:0005739)
Golgi membrane (GO:0000139)
cytoskeleton (GO:0005856)
Golgi subcompartment (GO:0098791)
nuclear body (GO:0016604)
integral component of plasma membrane (GO:0005887)
FRMD6
CUX2
GLB1L3
NMNAT3
DMGDH
PAK6
FBLL1
RASAL2
SNTG2
UPK2
CLCN1
Cellular Component
d)
Renal cell carcinoma
T cell receptor signaling pathway
Regulation of actin cytoskeleton
Axon guidance
PAK6
Human immunodeficiency virus 1 infection
ErbB signaling pathway
Focal adhesion
Ras signaling pathway
RASAL2
African trypanosomiasis
IDO2
Tryptophan metabolism
Sphingolipid signaling pathway
S1PR2
Neuroactive ligand-receptor interaction
Hippo signaling pathway
FRMD6
Hippo signaling pathway
NMNAT3
Nicotinate and nicotinamide metabolism
DMGDH
Glycine, serine and threonine metabolism
