# Supplementary material for: Implications of genetic variations, differential gene expression, and allele-specific expression on metformin response in drug-naïve type 2 diabetes
Source: J Endocrinol Invest. 2022 Dec 18;46(6):1205–18. doi: 10.1007/s40618-022-01989-y (PMC10185588; doi:10.1007/s40618-022-01989-y)
Supplement: Supplementary file 11 — Supplementary file11 (DOCX 58 KB) [file 40618_2022_1989_MOESM11_ESM.docx]

**Implications of genetic variations, differential gene expression, and allele-specific expression on metformin response in drug-naïve type 2 diabetes**

**Supplementary Table 1.** Coverage summary of designed targeted exome panel

| **Name** | **Chromosome** | **Amplicons** | **Total Bases** | **Covered Bases** | **Missed Bases** | **Overall Coverage** | **Exons Covered** |
| --- | --- | --- | --- | --- | --- | --- | --- |
| *ACACA* | chr17 | 57 | 7775 | 7775 | 0 | 1 | 59 |
| *ACACB* | chr12 | 57 | 7897 | 7792 | 105 | 0.987 | 52 |
| *ATM* | chr11 | 70 | 9791 | 9791 | 0 | 1 | 62 |
| *CRTC2* | chr1 | 16 | 2222 | 2222 | 0 | 1 | 14 |
| *CYP3A4* | chr7 | 14 | 1642 | 1642 | 0 | 1 | 14 |
| *GPAM* | chr10 | 22 | 2687 | 2687 | 0 | 1 | 20 |
| *HMGCR* | chr5 | 22 | 2857 | 2857 | 0 | 1 | 19 |
| *IRS1* | chr2 | 15 | 3739 | 3593 | 146 | 0.961 | 1 |
| *IRS2* | chr13 | 15 | 4037 | 3246 | 791 | 0.804 | 2 |
| *MLXIPL* | chr7 | 25 | 2729 | 2605 | 124 | 0.955 | 19 |
| *MLYCD* | chr16 | 9 | 1532 | 1493 | 39 | 0.975 | 5 |
| *MTOR* | chr1 | 58 | 8220 | 8220 | 0 | 1 | 57 |
| *NR1I2* | chr3 | 12 | 1512 | 1512 | 0 | 1 | 11 |
| *PPARGC1A* | chr4 | 16 | 2527 | 2527 | 0 | 1 | 13 |
| *RPTOR* | chr17 | 41 | 4348 | 4329 | 19 | 0.996 | 34 |
| *SIRT1* | chr10 | 12 | 2334 | 1967 | 367 | 0.843 | 10 |
| *SLC22A1* | chr6 | 14 | 1775 | 1775 | 0 | 1 | 12 |
| *SLC22A2* | chr6 | 13 | 1778 | 1778 | 0 | 1 | 11 |
| *SLC22A4* | chr5 | 11 | 1756 | 1756 | 0 | 1 | 10 |
| *SLC47A1* | chr17 | 18 | 1883 | 1878 | 5 | 0.997 | 17 |
| *SREBF1* | chr17 | 26 | 3734 | 3674 | 60 | 0.984 | 20 |
| *STK11* | chr19 | 14 | 1392 | 1392 | 0 | 1 | 9 |

**Supplementary Table 2.** The NGS run report from responders (n=13), non-responders (n=17) and healthy controls (n=15) samples

| **T2D Samples** | **Bases** | **≥Q20** | **Percentage Q20** | **Read** | **Mean Read Length** |
| --- | --- | --- | --- | --- | --- |
| MET10 | 15869820 | 14336567 | 90.34 | 68123 | 232 bp |
| MET11 | 77302193 | 67759242 | 87.66 | 351578 | 220 bp |
| MET13 | 64680073 | 56550768 | 87.43 | 294082 | 220 bp |
| MET16 | 30689908 | 27068922 | 88.20 | 148141 | 207 bp |
| MET17 | 24828659 | 21786581 | 87.75 | 111556 | 223 bp |
| MET20 | 60425403 | 52884007 | 87.52 | 276275 | 219 bp |
| MET21 | 92444293 | 83430397 | 90.25 | 397390 | 232 bp |
| MET22 | 5442578 | 4780892 | 87.84 | 25641 | 212 bp |
| MET24 | 19163289 | 17285068 | 90.20 | 77799 | 246 bp |
| MET3 | 45310373 | 39528458 | 87.24 | 212284 | 213 bp |
| MET31 | 19128741 | 16728303 | 87.45 | 86657 | 221 bp |
| MET32 | 30452457 | 26637954 | 87.47 | 143805 | 212 bp |
| MET33 | 51979918 | 45442082 | 87.42 | 239129 | 217 bp |
| MET35 | 49185883 | 44295376 | 90.06 | 200763 | 244 bp |
| MET37 | 76577514 | 66966799 | 87.45 | 348869 | 220 bp |
| MET38 | 56433938 | 49528102 | 87.76 | 273881 | 206 bp |
| MET4 | 61822305 | 55618537 | 89.97 | 254040 | 243 bp |
| MET41 | 17883424 | 16080931 | 89.92 | 75479 | 236 bp |
| MET44 | 66369910 | 60085719 | 90.53 | 297605 | 223 bp |
| MET46 | 79822333 | 72082579 | 90.30 | 327498 | 243 bp |
| MET49 | 62460416 | 56041449 | 89.72 | 256941 | 243 bp |
| MET50 | 49901447 | 44885913 | 89.95 | 205150 | 243 bp |
| MET54 | 36879166 | 33508008 | 90.86 | 167292 | 220 bp |
| MET55 | 13546261 | 12161653 | 89.78 | 55619 | 243 bp |
| MET56 | 26505021 | 24030907 | 90.67 | 119310 | 222 bp |
| MET57 | 5958441 | 5392329 | 90.50 | 25127 | 237 bp |
| MET6 | 52078405 | 45678795 | 87.71 | 235801 | 221 bp |
| MET7 | 74796119 | 65075782 | 87.00 | 344450 | 217 bp |
| MET8 | 11060958 | 9680076 | 87.52 | 51563 | 215 bp |
| MET9 | 23661492 | 20736011 | 87.64 | 107738 | 220 bp |
| Mean ± SD |  |  | 88.80 ± 1.35 |  | 225.67 ± 12.08 |
| **Control Samples** | **Bases** | **≥Q20** | **Percentage Q20** | **Read** | **Mean Read Length** |
| 3C | 39679881 | 37322969 | 94.06 | 149155 | 266 bp |
| 4C | 51186079 | 48501033 | 94.75 | 199603 | 256 bp |
| 5C | 61654414 | 58225772 | 94.44 | 235636 | 261 bp |
| 6C | 3674646 | 3483187 | 94.79 | 13927 | 263 bp |
| 7C | 107992142 | 101760190 | 94.23 | 409297 | 263 bp |
| 8C | 112836222 | 106465235 | 94.35 | 428926 | 263 bp |
| 11C | 30534335 | 28821418 | 94.39 | 113732 | 268 bp |
| 13C | 535179 | 504177 | 94.21 | 1999 | 267 bp |
| 14C | 8361130 | 7924971 | 94.78 | 33134 | 252 bp |
| 16C | 32878653 | 31108636 | 94.62 | 123022 | 267 bp |
| 28C | 72441415 | 68666733 | 94.79 | 287291 | 252 bp |
| 29C | 92233516 | 87100945 | 94.44 | 348841 | 264 bp |
| 30C | 46973292 | 44335368 | 94.38 | 178253 | 263 bp |
| 31C | 28382003 | 26816825 | 94.49 | 107122 | 264 bp |
| 32C | 50937186 | 48053896 | 94.34 | 194528 | 261 bp |
| 33C | 71996756 | 67877209 | 94.28 | 269833 | 266 bp |
| Mean ± SD |  |  | 94.48 ± 0.22 |  | 261.93 ± 4.94 |

**Supplementary Table 3.** Number of SNPs identified in the samples

| **S.No.** | **Hugo Symbol** | **Frame Shift Del** | **Frame Shift Ins** | **In Frame Del** | **Coding Mutation** | **Nonsense Mutation** | **Splice Site** | **total** | **Altered Samples** |
| --- | --- | --- | --- | --- | --- | --- | --- | --- | --- |
| 1 | *SLC22A1* | 12 | 4 | 4 | 109 | 0 | 0 | 129 | 45 |
| 2 | *ACACB* | 0 | 1 | 1 | 96 | 0 | 0 | 98 | 43 |
| 3 | *GPAM* | 0 | 0 | 0 | 82 | 0 | 0 | 82 | 43 |
| 4 | *ATM* | 1 | 1 | 0 | 61 | 0 | 0 | 63 | 43 |
| 5 | *SLC22A2* | 0 | 0 | 0 | 42 | 0 | 0 | 42 | 42 |
| 6 | *ACACA* | 3 | 0 | 0 | 50 | 0 | 0 | 53 | 40 |
| 7 | *PPARGC1A* | 0 | 0 | 0 | 33 | 2 | 0 | 35 | 31 |
| 8 | *CRTC2* | 0 | 0 | 0 | 33 | 0 | 0 | 33 | 29 |
| 9 | *IRS2* | 5 | 5 | 15 | 19 | 0 | 0 | 44 | 25 |
| 10 | *SLC22A4* | 0 | 1 | 0 | 38 | 0 | 0 | 39 | 25 |
| 11 | *MLXIPL* | 0 | 1 | 1 | 25 | 0 | 0 | 27 | 19 |
| 12 | *MTOR* | 1 | 1 | 1 | 15 | 0 | 0 | 18 | 11 |
| 13 | *SREBF1* | 2 | 0 | 0 | 9 | 0 | 0 | 11 | 10 |
| 14 | *IRS1* | 0 | 0 | 1 | 8 | 0 | 0 | 9 | 8 |
| 15 | *CYP3A4* | 0 | 0 | 0 | 4 | 1 | 1 | 6 | 6 |
| 16 | *NR1I2* | 0 | 0 | 1 | 4 | 0 | 0 | 5 | 4 |
| 17 | *STK11* | 1 | 0 | 0 | 4 | 0 | 0 | 5 | 4 |
| 18 | *MLYCD* | 0 | 0 | 0 | 4 | 0 | 0 | 4 | 4 |
| 19 | *SLC47A1* | 0 | 1 | 0 | 2 | 1 | 0 | 4 | 4 |
| 20 | *RPTOR* | 0 | 0 | 0 | 3 | 1 | 0 | 4 | 2 |
| 21 | *HMGCR* | 0 | 0 | 0 | 3 | 0 | 0 | 3 | 2 |
| 22 | *ABCC8* | 0 | 0 | 0 | 1 | 0 | 0 | 1 | 1 |

**Supplementary Table 4.** Association analysis for metformin drug response

| **Gene** | **Non-Responders** | **Responders** | **Odds ratio** | **CI_Upper limit** | **CI_Lower limit** | **p-value** |
| --- | --- | --- | --- | --- | --- | --- |
| *SLC22A4* | 6 | 11 | 0.10 | 0.73 | 0.008 | 0.01 |
| *SLC22A2* | 14 | 13 | 0 | 3.08 | 0 | 0.23 |
| *MLXIPL* | 9 | 4 | 2.45 | 15.55 | 0.44 | 0.28 |
| *ACACA* | 13 | 12 | 0.28 | 3.39 | 0.005 | 0.35 |
| *ACACB* | 15 | 13 | 0 | 6.95 | 0 | 0.49 |
| *ATM* | 15 | 13 | 0 | 6.95 | 0 | 0.49 |
| *IRS2* | 10 | 9 | 0.64 | 3.63 | 0.10 | 0.70 |
| *CRTC2* | 10 | 8 | 0.89 | 4.89 | 0.15 | 1 |
| *GPAM* | 16 | 13 | 0 | 50.96 | 0 | 1 |
| *PPARGC1A* | 11 | 8 | 1.14 | 6.48 | 0.19 | 1 |

**Supplementary Table 5.** Percentage of responders and non-responders samples showing copy number gain or loss

| **Regions showing copy number gain** | **Non-Responder (%)** | **Responder (%)** |
| --- | --- | --- |
| *IRS1* | 23.5 | 46.1 |
| *MLXIPL, CYP3A4* | 5.8 | 7.6 |
| *IRS2* | 47.05 | 30.7 |
| *MLYCD* | 17.6 | 23.07 |
| **Regions showing copy number loss** | **Non-Responder (%)** | **Responder (%)** |
| *NR1I2* | 5.8 | 7.6 |
| *PPARGC1A* | 17.6 | 0 |
| *HMGCR, SLC22A4* | 5.8 | 15.3 |
| *SIRT1, GPAM* | 5.8 | 7.6 |
| *ATM* | 5.8 | 7.6 |
| *ACACB* | 0 | 7.6 |
| *SREBF1, SLC47A1, ACACA, RPTOR* | 0 | 7.6 |
| *STK11* | 17.6 | 23.07 |

**Supplementary Table 6.** Comparison of protein coding genes identified in different groups

| S.no. | Group 1 | Category | Total number of genes | Unique Genes | Group 2 | Category | Total number of genes | Unique Genes | Common Genes |
| --- | --- | --- | --- | --- | --- | --- | --- | --- | --- |
| 1 | NR v/s R | Upregulated | 38 | 38 | R v/s C | Upregulated | 405 | 405 | 0 |
| 2 | NR v/s R | Upregulated | 38 | 9 | NR v/s C | Upregulated | 237 | 208 | 29* |
| 3 | R v/s C | Upregulated | 405 | 346 | NR v/s C | Upregulated | 237 | 178 | 59** |
| 4 | NR v/s R | Downregulated | 127 | 127 | R v/s C | Downregulated | 30 | 30 | 0 |
| 5 | NR v/s R | Downregulated | 127 | 127 | NR v/s C | Downregulated | 20 | 20 | 0 |
| 6 | R v/s C | Downregulated | 30 | 19 | NR v/s C | Downregulated | 20 | 9 | 11^‡^ |
| 7 | R v/s C | Upregulated | 405 | 405 | NR v/s C | Downregulated | 20 | 20 | 0 |
| 8 | NR v/s C | Upregulated | 237 | 237 | R v/s C | Downregulated | 30 | 30 | 0 |
| 9 | NR v/s R | Downregulated | 127 | 22 | R v/s C | Upregulated | 405 | 300 | 105^#^ |
| 10 | NR v/s R | Downregulated | 127 | 127 | NR v/s C | Upregulated | 237 | 237 | 0 |
| 11 | NR v/s R | Upregulated | 38 | 38 | R v/s C | Downregulated | 30 | 30 | 0 |
| 12 | NR v/s R | Upregulated | 38 | 38 | NR v/s C | Downregulated | 20 | 20 | 0 |

*NR, non-responder; R, responder and C, healthy control*

** Upregulate in non-responders when compared with responder or healthy controls (DR_Set_1)*

*** Upregulated in both non-responders and responders when compared healthy controls (T2D_Set_1)*

^‡^ *Downregulated in both non-responders and responders when compared healthy controls (T2D_Set_2)*

*# Upregulated in responders when compared with healthy controls but downregulated in non-responders when compared with responders (DR_Set_2)*

**Supplementary Table 7.** List of predicted markers for metformin non-response, response and T2D

| Genes | Full Form | Chromosome Coordinates | Function | Predicted Pathway | Predicted Group | Predicted Phenotype |
| --- | --- | --- | --- | --- | --- | --- |
| *IDO2* | Indoleamine 2,3-Dioxygenase 2 | chr8:39,934,614-40,016,391 | IDO2 (Indoleamine 2,3-Dioxygenase 2) is a Protein Coding gene. Among its related pathways are Histidine, lysine, phenylalanine, tyrosine, proline and tryptophan catabolism and superpathway of tryptophan utilization. Gene Ontology (GO) annotations related to this gene include heme binding and indoleamine 2,3-dioxygenase activity. | Tryptophan metabolism | NR vs R and NR vs C upregulated | Non-responder |
| *NMNAT3* | Nicotinamide Nucleotide Adenylyltransferase 3 | chr3:139,560,180-139,678,043 | This gene encodes a member of the nicotinamide/nicotinic acid mononucleotide adenylyltransferase family. These enzymes use ATP to catalyze the synthesis of nicotinamide adenine dinucleotide or nicotinic acid adenine dinucleotide from nicotinamide mononucleotide or nicotinic acid mononucleotide, respectively. | Nicotinate and nicotinamide metabolism | NR vs R and NR vs C upregulated | Non-responder |
| *FRMD6* | FERM Domain Containing 6 | chr14:51,489,100-51,730,727 | FRMD6 (FERM Domain Containing 6) is a Protein Coding gene. Among its related pathways are Hippo signaling pathway and Neuroscience. | Hippo Signalling Pathway | NR vs R and NR vs C upregulated | Non-responder |
| *DMGDH* | Dimethylglycine Dehydrogenase | chr5:78,997,564-79,236,038 | Catabolism of choline, catalyzing the oxidative demethylation of dimethylglycine to form sarcosine. The enzyme is found as a monomer in the mitochondrial matrix, and uses flavin adenine dinucleotide and folate as cofactors | Glycine, serine and threonone Metabolism | NR vs R and NR vs C upregulated | Non-responder |
| *RASAL2* | RAS Protein Activator Like 2 | chr1:178,093,729-178,484,147 | This gene encodes a protein that contains the GAP-related domain (GRD), a characteristic domain of GTPase-activating proteins (GAPs). GAPs function as activators of Ras superfamily of small GTPases | RAS signalling pathway | NR vs R and NR vs C upregulated | Non-responder |
| *S1PR2* | Sphingosine-1-Phosphate Receptor 2 | chr19:10,221,433-10,231,807 | The encoded protein is a receptor for sphingosine 1-phosphate, which participates in cell proliferation, survival, and transcriptional activation | Neuroactive ligand-receptor interaction | NR vs R and NR vs C upregulated | Non-responder |
| *PAK6* | P21 (RAC1) Activated Kinase 6 | chr15:40,217,428-40,277,487 | transcriptional regulation, cytoskeleton rearrangement, apoptosis, and the mitogen-activated protein (MAP) kinase signalling pathway | RAS signalling pathway | NR vs R and NR vs C upregulated | Non-responder |
| *GDF2* | Growth Differentiation Factor 2 | chr10:47,322,490-47,327,594 | GDF2 (Growth Differentiation Factor 2) is a Protein Coding gene. Among its related pathways are Apoptotic Pathways in Synovial Fibroblasts and GPCR Pathway. | Cytokine-cytokine receptor interaction | R vs C and NR vs C upregulated | T2D |
| *CXXC4* | CXXC Finger Protein 4 | chr4:104,468,306-104,494,901 | CXXC4 (CXXC Finger Protein 4) is a Protein Coding gene. Among its related pathways are WNT mediated activation of DVL and Signaling by Wnt | Wnt signaling pathway | R vs C and NR vs C upregulated | T2D |
| *MAGI2* | Membrane Associated Guanylate Kinase, WW And PDZ Domain Containing 2 | chr7:78,017,055-79,453,805 | MAGI2 (Membrane Associated Guanylate Kinase, WW And PDZ Domain Containing 2) is a Protein Coding gene. Among its related pathways are Nephrin interactions and PI3K-Akt signaling pathway | PI3K-Akt signaling pathway | R vs C and NR vs C upregulated | T2D |
| *MID1* | Midline 1 | chrX:10,445,310-11,111,177 | MID1 (Midline 1) is a Protein Coding gene. Among its related pathways are Cytokine Signaling in Immune system andInnate Immune System | Ubiquitin mediated proteolysis | R vs C and NR vs C upregulated | T2D |
| *CACNG8* | Calcium Voltage-Gated Channel Auxiliary Subunit Gamma 8 | chr19:53,963,036-53,990,215 | CACNG8 (Calcium Voltage-Gated Channel Auxiliary Subunit Gamma 8) is a Protein Coding gene. Among its related pathways are NFAT and Cardiac Hypertrophy and Fc-GammaR Pathway. | MAPK signaling pathway | R vs C and NR vs C upregulated | T2D |
| *CXADR* | CXADR, Ig-Like Cell Adhesion Molecule | chr21:17,512,382-17,633,323 | CXADR (CXADR, Ig-Like Cell Adhesion Molecule) is a Protein Coding gene. Among its related pathways are Class I MHC mediated antigen processing and presentation and Cell surface interactions at the vascular wall. | Viral myocarditis | R vs C and NR vs C upregulated | T2D |
| *HFE2* | Hemojuvelin BMP Co-Receptor | chr1:146,017,468-146,036,746 | Acts as a bone morphogenetic protein (BMP) coreceptor. | BMP receptor signaling | R vs C and NR vs C upregulated | T2D |
| *NDUFA4L2* | NDUFA4, Mitochondrial Complex Associated Like 2 | chr12:57,234,903-57,240,762 | NDUFA4L2 (NDUFA4, Mitochondrial Complex Associated Like 2) is a Protein Coding gene. Among its related pathways are Respiratory electron transport, ATP synthesis by chemiosmotic coupling, and heat production by uncoupling proteins. and GABAergic synapse | Oxidative phosphorylation | R vs C and NR vs C upregulated | T2D |
| *SOX4* | SRY (Sex Determining Region Y)-Box 4 | chr6:21,592,768-21,598,619 | SOX4 (SRY-Box 4) is a Protein Coding gene. Among its related pathways are MicroRNAs in cancer and Deactivation of the beta-catenin transactivating complex. | MicroRNAs in cancer | R vs C and NR vs C upregulated | T2D |
| *GBP5* | Guanylate Binding Protein 5 | chr1:89,258,950-89,272,861 | GBP5 (Guanylate Binding Protein 5) is a Protein Coding gene. Among its related pathways are Cytokine Signaling in Immune system andToll-like Receptor Signaling Pathway. | NOD-like receptor signaling pathway | R vs C and NR vs C downregulated | T2D |
| *P2RY14* | Purinergic Receptor P2Y14 | chr3:151,212,115-151,278,628 | P2RY14 (Purinergic Receptor P2Y14) is a Protein Coding gene. Among its related pathways are Nucleotide-like (purinergic) receptors and Peptide ligand-binding receptors | Neuroactive ligand-receptor interaction | R vs C and NR vs C downregulated | T2D |
| *LAMP3* | Lysosomal Associated Membrane Protein 3 | chr3:183,122,213-183,163,839 | LAMP3 (Lysosomal Associated Membrane Protein 3) is a Protein Coding gene. Among its related pathways are Lysosome. | Lysosome | R vs C and NR vs C downregulated | T2D |
| *IDO1* | Indoleamine 2,3-Dioxygenase 1 | chr8:39,902,275-39,928,790 | IDO1 (Indoleamine 2,3-Dioxygenase 1) is a Protein Coding gene. Among its related pathways are Histidine, lysine, phenylalanine, tyrosine, proline and tryptophan catabolism and NF-kappaB Signaling. | African trypanosomiasis | R vs C and NR vs C downregulated | T2D |
| *CXCL10* | C-X-C Motif Chemokine Ligand 10 | chr4:76,021,116-76,023,536 | CXCL10 (C-X-C Motif Chemokine Ligand 10) is a Protein Coding gene. Diseases associated with CXCL10 include Endotheliitis and Tafro Syndrome. Among its related pathways are Interleukin-10 signaling and TNF signaling pathway | Cytokine-cytokine receptor interaction | R vs C and NR vs C downregulated | T2D |
| *EIF5B* | Eukaryotic Translation Initiation Factor 5B | chr2:99,337,353-99,401,326 | EIF5B (Eukaryotic Translation Initiation Factor 5B) is a Protein Coding gene. Among its related pathways are Viral mRNA Translation and Translation Factors. | RNA transport | R vs C up and NR vs R down | Responder |
| *CD82* | CD82 Molecule | chr11:44,564,427-44,620,363 | CD82 (CD82 Molecule) is a Protein Coding gene. Among its related pathways are NF-kappaB Signaling and Direct p53 effectors | p53 signaling pathway | R vs C up and NR vs R down | Responder |
| *PRKAR2A* | Protein Kinase CAMP-Dependent Type II Regulatory Subunit Alpha | chr3:48,744,597-48,847,846 | PRKAR2A (Protein Kinase CAMP-Dependent Type II Regulatory Subunit Alpha) is a Protein Coding gene. Among its related pathways are Beta-Adrenergic Signaling and Signaling by Hedgehog | RNA transport | R vs C up and NR vs R down | Responder |
| *SEC62* | SEC62 Homolog, Preprotein Translocation Factor | chr3:169,966,635-169,998,373 | SEC62 (SEC62 Homolog, Preprotein Translocation Factor) is a Protein Coding gene. Among its related pathways are Unfolded Protein Response (UPR) and Protein processing in endoplasmic reticulum | Protein export | R vs C up and NR vs R down | Responder |
| *SNRPB2* | Small Nuclear Ribonucleoprotein Polypeptide B2 | chr20:16,729,961-16,742,563 | SNRPB2 (Small Nuclear Ribonucleoprotein Polypeptide B2) is a Protein Coding gene. Among its related pathways are mRNA Splicing - Major Pathway and Gene Expression | Spliceosome | R vs C up and NR vs R down | Responder |
| *MAP3K13* | Mitogen-Activated Protein Kinase Kinase Kinase 13 | chr3:185,282,941-185,489,097 | MAP3K13 (Mitogen-Activated Protein Kinase Kinase Kinase 13) is a Protein Coding gene. Among its related pathways are Integrated Breast Cancer Pathway and Angiopoietin Like Protein 8 Regulatory Pathway. | MAPK signaling pathway | R vs C up and NR vs R down | Responder |
| *RENBP* | Ribosomal Protein L21 | chr13:27,251,309-27,256,691 | RPL21 (Ribosomal Protein L21) is a Protein Coding gene. Among its related pathways are Viral mRNA Translation and Influenza Viral RNA Transcription and Replication | Amino sugar and nucletide sugar metabolism | R vs C up and NR vs R down | Responder |
| *RPL36A* | Ribosomal Protein L36a | chrX:101,390,824-101,396,154 | RPL36A (Ribosomal Protein L36a) is a Protein Coding gene. Among its related pathways are Viral mRNA Translation and Influenza Viral RNA Transcription and Replication. | Ribosome | R vs C up and NR vs R down | Responder |
| *RPL21* | Ribosomal Protein L21 | chr13:27,251,309-27,256,691 | RPL21 (Ribosomal Protein L21) is a Protein Coding gene. Among its related pathways are Viral mRNA Translation and Influenza Viral RNA Transcription and Replication. | Ribosome | R vs C up and NR vs R down | Responder |
| *DCTN6* | Dynactin Subunit 6 | chr8:30,156,297-30,183,640 | DCTN6 (Dynactin Subunit 6) is a Protein Coding gene. Among its related pathways are Metabolism of proteins and Golgi-to-ER retrograde transport. | Vasopressin-regulated water reabsorption | R vs C up and NR vs R down | Responder |
| *DAAM1* | Dishevelled Associated Activator Of Morphogenesis 1 | chr14:59,188,611-59,371,405 | DAAM1 (Dishevelled Associated Activator Of Morphogenesis 1) is a Protein Coding gene. Among its related pathways are Signaling by Rho GTPases and CDK-mediated phosphorylation and removal of Cdc6 | Wnt signaling pathway | R vs C up and NR vs R down | Responder |
| *TWISTNB* | TWIST Neighbor | chr7:19,695,462-19,709,087 | TWISTNB (TWIST Neighbor) is a Protein Coding gene. Among its related pathways are Activated PKN1 stimulates transcription of AR (androgen receptor) regulated genes KLK2 and KLK3 and RNA Polymerase III Transcription Initiation. | RNA polymerase | R vs C up and NR vs R down | Responder |
| *SLC16A10* | Solute Carrier Family 16 Member 10 | chr6:111,087,503-111,231,194 | SLC16A10 (Solute Carrier Family 16 Member 10) is a Protein Coding gene. Among its related pathways are Amino acid transport across the plasma membrane and Transport of glucose and other sugars, bile salts and organic acids, metal ions and amine compounds. | Thyroid hormone signaling pathway | R vs C up and NR vs R down | Responder |
| *P2RX7* | Purinergic Receptor P2X 7 | chr12:121,132,819-121,188,032 | P2RX7 (Purinergic Receptor P2X 7) is a Protein Coding gene. Among its related pathways are Toll-like Receptor Signaling Pathwayand CREB Pathway | Calcium signaling pathway | R vs C up and NR vs R down | Responder |
| *LIPT1* | Lipoyltransferase 1 | chr2:99,154,955-99,163,157 | LIPT1 (Lipoyltransferase 1) is a Protein Coding gene. Among its related pathways are Glyoxylate metabolism and glycine degradation and Viral mRNA Translation. | Lipoic acid metabolism | R vs C up and NR vs R down | Responder |
| *AK9* | Adenylate Kinase 9 | chr6:109,492,855-109,691,217 | AK9 (Adenylate Kinase 9) is a Protein Coding gene. Among its related pathways areSynthesis and interconversion of nucleotide di- and triphosphates and Purine metabolism (KEGG) | Purine metabolism | R vs C up and NR vs R down | Responder |
| *AGMAT* | Agmatinase | chr1:15,571,699-15,585,110 | AGMAT (Agmatinase) is a Protein Coding gene. Among its related pathways are CDK-mediated phosphorylation and removal of Cdc6 and Arginine and proline metabolism | Arginine and proline metabolism | R vs C up and NR vs R down | Responder |
| *RDH16* | Retinol Dehydrogenase 16 | chr12:56,951,431-56,959,374 | RDH16 (Retinol Dehydrogenase 16) is a Protein Coding gene. Among its related pathways are Drug metabolism - cytochrome P450 and Signaling by Retinoic Acid | Retinol metabolism | R vs C up and NR vs R down | Responder |
| *CD2AP* | CD2 Associated Protein | chr6:47,477,746-47,627,263 | CD2AP (CD2 Associated Protein) is a Protein Coding gene. Among its related pathways are Nephrin interactions and Signaling events mediated by VEGFR1 and VEGFR2 | Bacterial invasion of epithelial cells | R vs C up and NR vs R down | Responder |
| *SAV1* | Salvador Family WW Domain Containing Protein 1 | chr14:50,632,058-50,668,357 | SAV1 (Salvador Family WW Domain Containing Protein 1) is a Protein Coding gene. Among its related pathways are Hippo signaling pathway and Wnt / Hedgehog / Notch. | Hippo Signalling Pathway | R vs C up and NR vs R down | Responder |
| *SRD5A3* | Steroid 5 Alpha-Reductase 3 | chr4:55,346,109-55,373,100 | SRD5A3 (Steroid 5 Alpha-Reductase 3) is a Protein Coding gene. Among its related pathways are Metabolism of proteins and Synthesis of substrates in N-glycan biosythesis. | Steroid hormone biosynthesis | R vs C up and NR vs R down | Responder |
| *HBA2* | Hemoglobin Subunit Alpha 2 | chr16:172,847-173,710 | HBA2 (Hemoglobin Subunit Alpha 2) is a Protein Coding gene. Among its related pathways are Erythrocytes take up carbon dioxide and release oxygen and Binding and Uptake of Ligands by Scavenger Receptors | African trypanosomiasis | R vs C up and NR vs R down | Responder |
| *GDF15* | Growth Differentiation Factor 15 | chr19:18,374,731-18,389,176 | GDF15 (Growth Differentiation Factor 15) is a Protein Coding gene. Among its related pathways are Apoptotic Pathways in Synovial Fibroblasts and GPCR Pathway | Cytokine-cytokine receptor interaction | R vs C up and NR vs R down | Responder |
| *HBA1* | Hemoglobin Subunit Alpha 1 | chr16:176,651-177,522 | HBA1 (Hemoglobin Subunit Alpha 1) is a Protein Coding gene.Among its related pathways are Selenium Micronutrient Network and Erythrocytes take up carbon dioxide and release oxygen | African trypanosomiasis | R vs C up and NR vs R down | Responder |
| *POLR3G* | RNA Polymerase III Subunit G | chr5:90,471,748-90,514,677 | POLR3G (RNA Polymerase III Subunit G) is a Protein Coding gene. Among its related pathways are RNA Polymerase III Transcription Initiation and RIG-I/MDA5 mediated induction of IFN-alpha/beta pathways. | RNA polymerase | R vs C up and NR vs R down | Responder |
| *EIF4E* | Eukaryotic Translation Initiation Factor 4E | chr4:98,871,684-98,930,637 | The protein encoded by this gene is a component of the eukaryotic translation initiation factor 4F complex, which recognizes the 7-methylguanosine cap structure at the 5' end of messenger RNAs | RNA transport | R vs C up and NR vs R down | Responder |
| *OXTR* | Oxytocin Receptor | chr3:8,750,408-8,854,549 | The protein encoded by this gene belongs to the G-protein coupled receptor family and acts as a receptor for oxytocin | Calcium signaling pathway | R vs C up and NR vs R down | Responder |
| *HSD11B1* | Hydroxysteroid 11-Beta Dehydrogenase 1 | chr1:209,686,178-209,734,950 | The protein encoded by this gene is a microsomal enzyme that catalyzes the conversion of the stress hormone cortisol to the inactive metabolite cortisone. In addition, the encoded protein can catalyze the reverse reaction, the conversion of cortisone to cortisol | Steroid hormone biosynthesis | R vs C up and NR vs R down | Responder |

**Supplementary Table 8.** Interaction of differentially expressed genes with other drugs

| Gene | Drug | Interaction  Types | Sources | PMIDs |
| --- | --- | --- | --- | --- |
| *NMNAT3* | Betanmn | - | DrugBank | 17139284; 17016423 |
| *NMNAT3* | Diphosphomethylphosphonic Acid Adenosyl Ester | - | DrugBank | 17139284; 17016423 |
| *RENBP* | N-Acetylglucosamine | - | DrugBank | 17292397; 17349707; 17565386; 16011304 |
| *LIPT1* | Lipoic Acid | - | DrugBank | 16043486; 8617275; 17570395; 16246025; 10473591 |
| *GDF15* | Sulindac Sulfide | - | NCI | 15180942 |
| *GDF15* | Diclofenac | - | NCI | 15555568 |
| *GDF15* | Etoposide | - | NCI | 10618379 |
| *GDF15* | Indole-3-Carbinol | - | NCI | 15670751 |
| *GDF15* | Resveratrol | - | NCI | 11895857 |
| *GDF15* | Calcitriol | - | NCI | 15386353 |
| *HBA1* | Iron Dextran | activator | DrugBank | 11752352; 17139284; 17016423 |
| *HBA1* | Sebacic Acid | - | DrugBank | 10592235 |
| *HBA1* | Efaproxiral | - | DrugBank | 10592235 |
| *EIF4E* | Hydrogen Peroxide | - | NCI | 12151318 |
| *EIF4E* | Sirolimus | - | NCI | 10516161; 8816458; 10477262; 15292274; 14581487; 9867830 |
| *EIF4E* | Etoposide | - | NCI | 11956083 |
| *EIF4E* | Hydroxyurea | - | NCI | 10585489 |
| *OXTR* | Oxytocin | agonist | DrugBank | 21208749; 20108008; 18655883; 20104850; 15646817 |
| *OXTR* | Carbetocin | agonist | DrugBank | 15740719; 11752352; 9760035 |
| *OXTR* | Progesterone | - | NCI | 12061859 |
| *OXTR* | Estradiol | - | NCI | 3033233 |
| *OXTR* | Sodium Chloride | - | NCI | 12907413 |
| *OXTR* | Testosterone | - | NCI | 1846593 |
| *HSD11B1* | Prednisone | ligand | DrugBank | 20634231 |
| *HSD11B1* | Carbenoxolone | inhibitor | DrugBank | 11752352 |
| *HSD11B1* | Corticosterone | - | DrugBank | 10592235 |

NCI; National Cancer Institute

**Supplementary Table 9.** Allele specific expression observed in control, responder and non-responder samples

| **Gene** | **dbSNP** | **Type** | **MAF** | **Average Allelic Ratio** | | |
| --- | --- | --- | --- | --- | --- | --- |
|  |  |  |  | **Control** | **Responder** | **Non-Responder** |
| *IRS2* | rs4773092 | synonymous | 0.43 | 0.43 | 0.60 | 0.15 |
| *ACACB* | rs2075262 | 3'UTR variant | 0.35 | 0.39 | 0.43 | 0.54 |
| *CRTC2* | rs11264680 | missense | 0.26 | 0.32 | 0.29 | 0.28 |
| *CRTC2* | rs10559 | 3'UTR variant | 0.26 | 0.37 | 0.40 | 0.37 |
| *MTOR* | rs1135172 | synonymous | 0.36 | 0.62 | 0.53 | 0.76 |
| *MTOR* | rs1057079 | synonymous | 0.45 | 0.64 | 0.29 | 0.51 |
| *RPTOR* | rs1567962 | synonymous | 0.37 | 0.72 | 0.82 | 0.64 |
| *RPTOR* | rs2289764 | synonymous | 0.38 | 0.40 | 0.78 | 0.44 |
| *SLC22A4* | rs272879 | synonymous | 0.47 | 0.46 | 0.60 | 0.00 |
