# Supplementary material for: Implications of genetic variations, differential gene expression, and allele-specific expression on metformin response in drug-naïve type 2 diabetes
Source: J Endocrinol Invest. 2022 Dec 18;46(6):1205–18. doi: 10.1007/s40618-022-01989-y (PMC10185588; doi:10.1007/s40618-022-01989-y)
Supplement: Supplementary file 8 — Supplementary Fig. 7. Gene Ontology (GO) analysis of genes in Metformin_Set_2. (a) Biological process, (b) Molecular function, (c) Cellular component and (d) Enrichment for GO groups (PDF 394 KB) [file 40618_2022_1989_MOESM8_ESM.pdf]

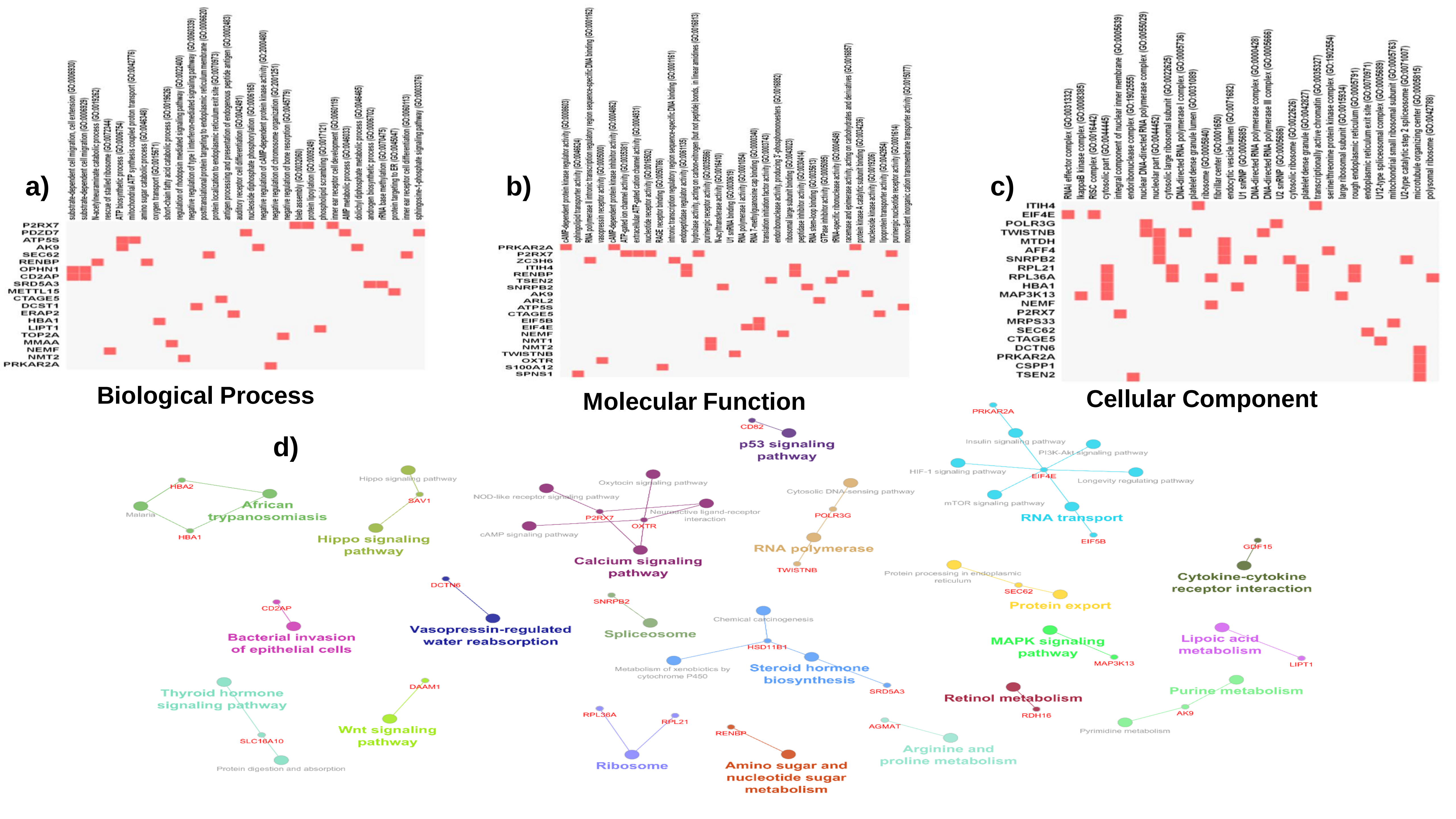

a)
P2RX7
PDZD7
ATP5S
AK9
SEC62
RENBP
OPHN1
CD2AP
SRD5A3
METTL15
CTAGE5
DCST1
ERAP2
HBA1
LIPT1
TOP2A
MMAA
NEMF
NMT2
PRKAR2A
Biological Process
b)
PRKAR2A
P2RX7
ZC3H6
ITIH4
RENBP
TSEN2
SNRPB2
AK9
ARL2
ATP5S
CTAGE5
EIF5B
EIF4E
NEMF
NMT1
NMT2
TWISTNB
OXTR
S100A12
SPNS1
Molecular Function
c)
ITIH4
EIF4E
POLR3G
TWISTNB
MTDH
AFF4
SNRPB2
RPL21
RPL36A
HBA1
MAP3K13
NEMF
P2RX7
MRPS33
SEC62
CTAGE5
DCTN6
PRKAR2A
CSPP1
TSEN2
Cellular Component
d)
HBA2
Malaria
African trypanosomiasis
HBA1
Hippo signaling pathway
SAV1
Hippo signaling pathway
Oxytocin signaling pathway
NOD-like receptor signaling pathway
P2RX7
Neuroactive ligand-receptor interaction
OXTR
cAMP signaling pathway
Calcium signaling pathway
CD82
p53 signaling pathway
Cytosolic DNA-sensing pathway
POLR3G
RNA polymerase
TWISTNB
PRKAR2A
Insulin signaling pathway
PI3K-Akt signaling pathway
HIF-1 signaling pathway
EIF4E
Longevity regulating pathway
mTOR signaling pathway
RNA transport
EIF5B
GDF15
Cytokine-cytokine receptor interaction
Protein processing in endoplasmic reticulum
SEC62
Protein export
CD2AP
Bacterial invasion of epithelial cells
DCTN6
Vasopressin-regulated water reabsorption
SNRPB2
Spliceosome
Chemical carcinogenesis
HSD11B1
Metabolism of xenobiotics by cytochrome P450
Steroid hormone biosynthesis
SRD5A3
MAPK signaling pathway
MAP3K13
Lipoic acid metabolism
LIPT1
Thyroid hormone signaling pathway
SLC16A10
Protein digestion and absorption
DAAM1
Wnt signaling pathway
RPL36A
RPL21
Ribosome
RENBP
Amino sugar and nucleotide sugar metabolism
AGMAT
Arginine and proline metabolism
Retinol metabolism
RDH16
Purine metabolism
AK9
Pyrimidine metabolism
